# Supplementary material for: The effect of mowing and mulching on snail communities: an experiment in wet meadows
Source: PLoS One. 2025 Jul 10;20(7):e0314670. doi: 10.1371/journal.pone.0314670 (PMC12244540; doi:10.1371/journal.pone.0314670)
Supplement: S1 Table — (DOCX) [file pone.0314670.s001.docx]

|  | Pretreatment | | | Posttreatment | | |
| --- | --- | --- | --- | --- | --- | --- |
|  | Control | Mowed | Mulched | Control | Mowed | Mulched |
| Szilvásvárad | 260 | 280 | 193 | 239 | 117 | 253 |
| Tarnalelesz | 559 | 903 | 877 | 447 | 510 | 595 |
