# Supplementary material for: The effect of mowing and mulching on snail communities: an experiment in wet meadows
Source: PLoS One. 2025 Jul 10;20(7):e0314670. doi: 10.1371/journal.pone.0314670 (PMC12244540; doi:10.1371/journal.pone.0314670)
Supplement: S2 Table — Posthoc tests from GLMMs separately fitted to each response variable (see the main text for details). (DOCX) [file pone.0314670.s002.docx]

|  | Pretreatment | | |
| --- | --- | --- | --- |
| Response variable | Control-Mowed | Control-Mulched | Mowed-Mulched |
| Number of live specimens | z = -1.39, p = 0.35 | z = -0.66, p = 0.79 | z = 0.75, p = 0.73 |
| Number of species | t = -2.20, p = 0.07 | t = -0.27, p = 0.96 | t = 1.92, p = 0.14 |
| Evenness | t = 0.50, p = 0.87 | t = -0.56, p = 0.84 | t = -1.09, p = 0.52 |
