# Supplementary material for: The effect of mowing and mulching on snail communities: an experiment in wet meadows
Source: PLoS One. 2025 Jul 10;20(7):e0314670. doi: 10.1371/journal.pone.0314670 (PMC12244540; doi:10.1371/journal.pone.0314670)
Supplement: S3 Table — Posthoc tests from GLMMs separately fitted to each response variable (see the main text for details). Legend: Bold font indicates significant differences. (DOCX) [file pone.0314670.s003.docx]

| Response variable | Control | Mowed | Mulched |
| --- | --- | --- | --- |
| Number of live specimens (pre-/post-treatment) | z = 1.62, p = 0.10 | **z = 4.68, p < 0.0001** | z = 1.43, p = 0.15 |
| Number of species (pre-/post-treatment) | t = 1.53, p = 0.13 | **t = 5.86, p < 0.0001** | t = 1.62, p = 0.11 |
| Evenness (pre-/post-treatment) | t = -0.31, p = 0.76 | **t = -2.39, p = 0.02** | t = 1.17, p = 0.24 |
