# Supplementary material for: The effect of mowing and mulching on snail communities: an experiment in wet meadows
Source: PLoS One. 2025 Jul 10;20(7):e0314670. doi: 10.1371/journal.pone.0314670 (PMC12244540; doi:10.1371/journal.pone.0314670)
Supplement: Attachment — Submitted filename: PONE-D-24-52289_R1_reviewer_incl_reviewer_remarks.pdf [file pone.0314670.s006.pdf]

PLOS ONE

The effect of mowing and mulching on snail communities: an experiment in wet meadows

--Manuscript Draft--

**Manuscript Number:**	PONE-D-24-52289R1
**Article Type:**	Research Article
**Full Title:**	The effect of mowing and mulching on snail communities: an experiment in wet meadows
**Short Title:**	Experimental comparison of mowing and mulching on snail communities in wet meadows
**Corresponding Author:**	Roland Farkas<br>Bükk National Park Directorate<br>Eger, HUNGARY
**Keywords:**	Gastropoda; biodiversity; abundance; Management; Conservation
**Abstract:**	The condition of wet meadows nowadays depends mainly on human activities; the biodiversity and productivity of these habitats can only be maintained through appropriate management methods. Mowing and grazing are well-known traditional methods, but a new method, regular mulching – the shredding of plant material and its deposition in the area - is becoming more widely practised. To better understand the impact of these methods we directly compared their effects on snails, an invertebrate taxon common in these habitats. We experimentally manipulated two wet meadows in Northern Hungary, Europe, and surveyed their snail communities immediately before and fourteen months after treatment. Our results showed that mowing had a detectable negative effect on the snail communities, whilst mulching did not alter their characteristics. Therefore, mulching may be a promising candidate for wet meadow habitats.
**Order of Authors:**	Roland Farkas
	Miklós Bán
	Gergő Oláh
	György Dudás
	Zoltán Barta
**Response to Reviewers:**	Dear Reviewers,<br><br>I would like to thank you for your dedicated work, which contributed to the development of our manuscript. We found all your questions and suggestions valuable. We have done our best to revise the manuscript with your help in accordance with the professional needs of the scientific field and the journal. We especially thank you for your patience and understanding regarding the linguistic shortcomings, upon your suggestion we had it checked by a native speaker. We have responded to all your questions and suggestions, which you can find in the uploaded rebuttal letter. We thank you once again for your work and wish you further success in your professional career.<br><br>Additional Editor Comments:<br>The study's limited scale (two sites, three treatments) necessitates a careful acknowledgement of its constraints.<br>Thank you for your comment, we have included your suggestion in the Discussion chapter.<br>Include limitations of the study and suggestions for future research, particularly regarding long-term effects and additional taxa.<br>We have made the requested additions int he Discussion chapter..<br>Provide precise details about the mowing and mulching machinery used (e.g., bar mower, disc mower, or flail mulcher).<br>The parameters were defined in the Method section upon your request.

Powered by Editorial Manager® and ProduXion Manager® from Aries Systems Corporation

Address awkward phrasing and inconsistent terminology (e.g., replace "parcel" with "plot," and "vegetable origin" with "organic material").
Based on your and the reviewers' helpful and detailed suggestions, we have made several modifications to the wording of our manuscript. We are confident that the readability of the manuscript has improved.
Differentiate between immediate and longer-term effects of management practices and contextualize results within this framework.
We have increased the elaboration of the question in both the Introduction and the Discussion chapters.

[Note: HTML markup is below. Please do not edit.]

Reviewers' comments:

Reviewer's Responses to Questions
Comments to the Author

1. Is the manuscript technically sound, and do the data support the conclusions?

The manuscript must describe a technically sound piece of scientific research with data that supports the conclusions. Experiments must have been conducted rigorously, with appropriate controls, replication, and sample sizes. The conclusions must be drawn appropriately based on the data presented.
Reviewer #1: Yes
Reviewer #2: Yes

2. Has the statistical analysis been performed appropriately and rigorously?
Reviewer #1: Yes
Reviewer #2: Yes

3. Have the authors made all data underlying the findings in their manuscript fully available?

The PLOS Data policy requires authors to make all data underlying the findings described in their manuscript fully available without restriction, with rare exception (please refer to the Data Availability Statement in the manuscript PDF file). The data should be provided as part of the manuscript or its supporting information, or deposited to a public repository. For example, in addition to summary statistics, the data points behind means, medians and variance measures should be available. If there are restrictions on publicly sharing data—e.g. participant privacy or use of data from a third party—those must be specified.
Reviewer #1: Yes
Reviewer #2: No

4. Is the manuscript presented in an intelligible fashion and written in standard English?

PLOS ONE does not copyedit accepted manuscripts, so the language in submitted articles must be clear, correct, and unambiguous. Any typographical or grammatical errors should be corrected at revision, so please note any specific errors here.
Reviewer #1: No
Reviewer #2: Yes

5. Review Comments to the Author

Please use the space provided to explain your answers to the questions above. You may also include additional comments for the author, including concerns about dual publication, research ethics, or publication ethics. (Please upload your review as an attachment if it exceeds 20,000 characters)
Reviewer #1: This study investigates the effects of two different grassland management techniques—mowing and mulching—on mollusk communities in two wet meadow sites. The aim was to determine which management practice would have the least detrimental impact on these communities. While not explicitly stated, the authors anticipated that the management practices would have varying effects on the number

of live mollusk specimens, species richness, and Shannon diversity. The authors found that mowing have significant effect on the mollusk communities: mowing reduced the number of living individuals and number of species, but increased Shannon diversity. Mulching had no significant effect. The study concludes that grassland management practices can alter mollusk communities in wet meadows, highlighting the importance of considering these impacts when choosing management strategies.

The authors' experimental design for this field study is commendable. Despite the small scale, the research addresses a relevant question regarding conservation management, particularly given the scarcity of literature on the effects of mulching on grassland fauna. A key weakness of the study design is the inconsistency in sampling times between years. While pre-treatment data were collected in June, post-treatment data were collected in August of the following year.
Thank you for your comment. The pre-management data collection, mechanical management and post-treatment data collection were performed simultaneously in the control, mowed and mulched plots. Consequently, the same amount of time elapsed between each action in all three treatment plots, therefore we believe that the results of our tests (a significant interaction between treatment and sampling period) were not affected by the fact that it covered a period of 14 months rather than a calendar year. We agree that the terms "short term" or "one year" used for the duration of our work are not accurate, so we have corrected this in the manuscript.

The study's short-term before-after design, without subsequent monitoring in the years following treatment application, restricts its ability to assess long-term community responses and potential delayed effects of mowing and mulching.
Our design was specifically to examine the short-term effects of treatments. We tried to avoid to draw long-term conclusions based on our results. Changes occurring in the short term may help to identify adverse processes early, which allows for the transition to other, more appropriate methods in sensitive areas. We also described this idea in the manuscript.
While the manuscript generally meets the formal requirements of a scientific paper, the language requires substantial improvement. Numerous typos, awkward phrasing, and occasional illogical sentence structures suggest potential translation issues.

We are very grateful that you devoted so much energy to improving the language of our manuscript! Your suggestions meant a lot to us, so we used them in almost every case to improve the manuscript. We also asked a fluent English speaker to improve the language of our MS.

Abstract:
line19: ...maintained through appropriate management method …
line21: shredding, instead of fragmentation
line22: ...is becoming more widely practiced. Or something similar
line22-23: Informal. It could be replaced with more precise academic language.
line30-31: ...while also preserving their biodiversity.

Introduction:
line42: use in-text reference consistently. Suggestion: Squires et al. 2018
line40-41: The phrase "necessary for, first the nomadic lifestyle of livestock grazing" is awkwardly phrased and could be restructured for smoother readability.
line43-44: suggest: since the agricultural revolution, primarily due to human activities
line62: utilizing it, rather than farming
line64-65: Rephrase this sentence, expressions and grammar!
Line73-74: Consider breaking it into two sentences for clarity.
Line76-78: I do not think such explanation is needed for the practices.
Line79-81: Suggestion: The encroachment of shrubs and woody plants threatens the persistence of grasslands and can also negatively impact farming activities.
Line81: A striking example of these changes can be seen in Central and Eastern Europe…
Line85: use ; in the parentheses between 2 references
Line86: consistency in in-text references still needed
Line88: definitions, rather than meanings
Line96: lawn is a wrong translation of grassland/meadow
Line100: rather an area within a few years

Powered by Editorial Manager® and ProduXion Manager® from Aries Systems Corporation

Line109: „Less favourable temperatures" could be more specific, mentioning higher temperatures
Line114: Organic material/Biomass
Line132: ...has been less extensively studied.
Line124: do not use the word „farmed” in this context
Line151-152: The phrase "At most, conclusions can be drawn from experience with one kind of mulching" sounds awkward and informal.
Line155: populations can’t be indicator species
Line156: "to say after the first year" could be more formal
Line158-159: Suggest: We selected undisturbed wet meadows that had remained untouched for decades and characterized their snail communities. We then mowed and mulched portions of these areas using standard agricultural machinery, following a stratified experimental design.

In the aims section, you could be more precise, mention at least hte factors, which you use to assess how „harmful” these managements are. Hypotheses could be clarified as well.
Thank you for your comment, we have improved the sections indicated above according to your request.

Materials and methods:
Line175: I would avoid the phrasing „without any obvious microhabitat structure”, as microhabitat structure depends of the definition. Be precise.
Line177: „for the last 20-30 years” 10 years matters a lot!
Line180: Figure 1.: In my opinion, the two smaller maps below are not very informative. Consider replacing them with a different figure. It's important to show the location within Hungary, but additionally, it might be useful to show Hungary's location within Europe, rather than using two other small-scale maps which shows nothing. Including satellite images to illustrate vegetation coverage would be helpful, and adding some toponyms could assist those who want to identify the study sites. Alternatively, you could completely reorganize the figure. Besides the map of Hungary, photographs of the meadows might be more useful for better understanding.
We have transformed the figure. We considered the photographic solution to be appropriate, which was indeed more informative than the original. We solved the identification of the locations using coordinates given in the Method section.

Line196: It would be more obvious to show 45 sampling points on Figure 2, rather than a grid.

The figure has been modified and incorporated into Figure 1.
Line204: don’t need „once”
Line207-208: I think the aouthors did not mimic the traditional mowing of hay this way. The cut plant material remaines on the are for some days for drying. This is a totally different effect on snails, then removing the plant material immediately.

Thank you for your comment. To clarify the issue, we have explained the difference between the treatment methods we use and the traditional ones in the Discussion section. It is interesting that the articles based on mowing experiments only mention the fact of "mowing". After cutting the vegetation, they do not detail how many sub-steps were performed (drying, turning, windrowing, baling, etc.) until the cut biomass was removed. Perhaps more emphasis should be placed on detailing these in future studies. In our modification we also highlighted that management practice of nature conservation areas by mowing resembles more to our treatment than traditional hay producing mowing.
Line222: „vegetable origin”:)
Line241: don’t need „interactive”
Line253: don’t need „however”

Results:
Line276: scientific names should be italic in Table 1
Line292: authors may use Shannon diversity consitently
Line305-306: indexing χ21 χ22 χ22
On Figure 3c, relocate y axis title

Powered by Editorial Manager® and ProduXion Manager® from Aries Systems Corporation

Discussion:
Line328: check this sentence
Line 389: there is in additional comma in the reference parentheses

I would like to read some limitations, possible future prospects in the discussion. This part was much better formulated than the other parts of the article.
The indicated parts were corrected in the manuscript at your request.

Conclusion:
Line403: avoid such strong emotional phrases like „clearly evidendt"
Line405-406: Be cautious with the word „ptactical". Highlight the exact result like:...without the negative effects of mowing on snail communities
Line407: „to maintain" and „to preserve" would fit better
Line407-408: Link it more directly to your findings. Like: „Given the observed negative effects of mowing…"
Line413-415: This is too general. Connect it to the specific ecological aspects revealed by the study.

We modified this part at your request.

Literature:
Again, be consistent. Corrected author initials spacing (e.g., "KJ." → "K. J."), it is used multiple ways.
Thank you for your comment. We checked and corrected spacings throught the whole manuscript.

Appendix 2: Format the first column, odd spacing.
We modified this part at your request.

Reviewer #2: The manuscript „ Experimental comparison of mowing and mulching on snail communities in wet meadows" by Farkas et al. studied the effect of two common grassland management methods on the number of individuals, the species numbers and the diversity (Shannon-Index) of snails in wet meadows in Hungary one year after the treatment.
While mulching, the shredding of vegetation and leaving it on the ground, had no effect, mowing reduced the number of individuals and species, but increased the Shannon-diversity. From the most abundant species, only one was significantly affected by the treatment, with an increase in the number of individuals after mulching.
Technically, the study seems to be sound and the results seem to be well supported by the data. Samples were collected with appropriate methods and the data were analysed using up-to-date statistical methods. Therefore, considering the lack of data on the effect of intensive agriculture on snails in the face of the current biodiversity crisis the data certainly deserve publication.
My main concern is that the analysis of data is a bit meager and that the study is relatively small and consisted of one experiment only with three treatments (control, mowing, mulching) on two sites. While the first aspect can be addressed by some more statistical analyses (see remarks below), the second cannot be changed. Therefore, I would leave it to the editor to decide if this is sufficient to accept the manuscript for publication. Apart from this, I have several aspects that should be addressed to make the manuscript suitable for publication.
We thanks the reviewer effort on our MS and reply her/his detailed comments below.

Detailed comments
1. While it is interesting that only mowing affected individual numbers, species numbers and Shannon-diversity, it would be interesting to know the underlying reasons. This is the case for the demonstrated effects of mowing, but also the absence of any effect of mulching.
We now highlight some reasons why mowing had and mulching had not have an effect on snail communities. These are basically connected to the fact that after mowing all cut material was removed from the area, while after mulching the material remained which prevented the immediate desiccation of the area.

For instance, the unexpected rise in the Shannon-diversity by mowing is explained by the authors by a potential decline of individual numbers of abundant species and the

Powered by Editorial Manager® and ProduXion Manager® from Aries Systems Corporation

associated increase of evenness.
Here we have to point out a mistake we made in the previous version of the MS: We actually calculated Pileou’s evenness index for our samples which we incorrectly called the Shannon diversity. We now corrected this naming error throughout the MS.
This is an interesting hypothesis which could (and should) be tested by the authors with their data. First, authors should calculate the evenness for their samples. In addition, a closer look at the data should reveal whether there are in fact abundant species which have decreased in individual numbers.
We calculated the decrease in abundance as a function of original (pretreatment) abundance and got a negative relationship between the two in each treatment. Unfortunately, the distributional properties of our data prevented us to statistically test if this negative slope was steeper for the mowing than for the other two treatments. Therefore, we just present our idea as a hypothesis in the Discussion.
Likewise, it would be interesting to know, whether mulching really had no impact on the snail community (as suggested by the numbers of individuals and species), or whether there might have been a species turn-over between the plots before and after the treatment. This could be analysed using beta-diversity and a similarity analysis. The same analysis should be done with the data from the mown plots. In addition, authors should also look at the species lists from the different treatments and the ecology of species that have changed in abundance. In conclusion, a more detailed analysis of the data could over insights into the mechanisms which are responsible for the observed effects.
Thank you for this suggestion. We took a broader approach in the current version of the MS and use PERMANOVA (adonis2 function of the vegan R package) to test if our terms (treatment, sampling and their interaction) have any effect on the overal compositions of the differently treated plots. We got the same result as previously, namely we have a significant interaction between treatment and sampling which indicates that the communities reacted differently to the different treatments over the sampling period. Examing the estimated parameters of the PERMANOVA indicates that this differences only significant in the case of mowing in accordance with our previous results.

2. While it is correct that the authors have included “site” as random variable in their model, it would be also interesting to learn whether the observed effects occur equally on both sites. Therefore, I suggest an analysis in which site is used as fixed factor.
We repeated all of our analyses with including site as a fixed factor and none of our conclusion changed so we retained the original analyses.

3. Generally, the manuscript is well readable but not very well written and, in some parts, not very stringent and wordy. This is especially true for the introduction and the discussion. Also, the English sometimes seems wrong to me. For instance, the term “parcel” is used for the German term “Parzelle”. I think the correct translation should be “plots”. I have made a number of suggestions in the attached pdf-File, but I am not a native speaker. Therefore, I strongly recommend proof reading of the manuscript by a native speaker experienced in scientific writing before submission of the revised version.
At your request, we had the manuscript re-checked for grammatical accuracy by a professional English-speaking researcher.
4. Authors should not refer to snails as indicator species in the context of the study. As mentioned by the authors themselves, different taxa react very differently to agricultural management measures. Snaily might be indicators for themselves, but not for other groups!
Thank you for your comment, we have made the necessary changes in the Intoduction chapter.

5. Authors should be more specific with respect to the effect of mowing or mulching on the target organisms. This refers to line 133 of the introduction, but also to the rest of the manuscript. In the available literature, the effect of mowings has been studied immediately after the treatment, sometime after the treatment (weeks or years), or during longer periods of time. While effects found immediately after the treatment have to (and can) be attributed directly to the mowing event itself, i.e. the mortality caused by the mowing machine, effects after weeks or years can be increasingly indirect, e.g. increased mortality due to unfavourable environmental conditions or increased predation of exposed organisms. Authors should explicitly point out that their study

Powered by Editorial Manager® and ProduXion Manager® from Aries Systems Corporation

	refers to the second effects<br>We modified this part in the Introduction and Discussion chapters.<br>6. On how many replicates per treatement are the data based. Is it n=15 per site, i.e. n=30 per treatment for the whole study?<br>We have now clarified it in the Methods section, we have 30 replicate per treatment.<br><br>7. Throughout the ms: Tables description should be on top of the table.<br>We have modified it.<br>Line 93ff: What is the difference between these two variants of mulching. To me, it sounds identical. Grass is mown and left on the spot. Or is the fact that grass is shredded in variant 3, but not in variant 2?<br>Thank you for your comment, we made it clear in the Introduction chapter.<br><br>Line 207: Please include the type of mowing machine used? Bar mower, Disc mover Flail mulcher? Conditioner?<br>We have clarified it in the Method chapter.<br><br>Table 2: Use italics for species and genus names<br>We have modified it in all cases<br><br><br>6. PLOS authors have the option to publish the peer review history of their article (what does this mean?). If published, this will include your full peer review and any attached files.<br><br><br>If you choose "no", your identity will remain anonymous but your review may still be made public.<br><br><br>Do you want your identity to be public for this peer review? For information about this choice, including consent withdrawal, please see our Privacy Policy.<br>Reviewer #1: No<br>Reviewer #2: Yes: Johannes Steidle
**Additional Information:**	
**Question**	**Response**
**Financial Disclosure**<br><br>Enter a financial disclosure statement that describes the sources of funding for the work included in this submission. Review the submission guidelines for detailed requirements. View published research articles from *PLOS ONE* for specific examples.<br><br><br>This statement is required for submission and **will appear in the published article** if the submission is accepted. Please make sure it is accurate.	Yes

**Funded studies**<br>Enter a statement with the following details:<br>• Initials of the authors who received each award<br>• Grant numbers awarded to each author<br>• The full name of each funder<br>• URL of each funder website<br>• Did the sponsors or funders play any role in the study design, data collection and analysis, decision to publish, or preparation of the manuscript?<br><br>Did you receive funding for this work?	
Please add funding details.<br>as follow-up to "**Financial Disclosure**<br><br>Enter a financial disclosure statement that describes the sources of funding for the work included in this submission. Review the submission guidelines for detailed requirements. View published research articles from *PLOS ONE* for specific examples.<br><br>This statement is required for submission and **will appear in the published article** if the submission is accepted. Please make sure it is accurate.<br><br>**Funded studies**<br>Enter a statement with the following details:<br>• Initials of the authors who received each award<br>• Grant numbers awarded to each author<br>• The full name of each funder<br>• URL of each funder website<br>• Did the sponsors or funders play any role in the study design, data collection and analysis, decision to publish, or preparation of the manuscript?<br><br>Did you receive funding for this work?"	This was prepared with the professional support of the Doctoral Student Scholarship Program of the Co-operative Doctoral Program of the Ministry of Innovation and Technology financed from the National Research, Development and Innovation Fund (Hungary). The study was supported by a National Research, Development and Innovation Office grant (NKFIH K138503, https://nkfih.gov.hu). Zoltán Barta was supported by National Research, Development and Innovation Fund grants (TKP2021-NKTA-32 and K138503, https://nkfih.gov.hu) (Hungary). Gergő Oláh was supported by the EKÖP-24-3-I-DE-131 University Research Scholaship Program of the Ministry for Culture and Innovation from the source of the National Research, Development and Innovation Fund (RH/1094-173/2024, https://kormany.hu/kulturalis-es-innovacios-miniszterium). The funders had no role in study design, data collection and analysis, decision to publish, or preparation of the manuscript.
Please select the country of your main research funder (please select carefully as in some cases this is used in fee calculation).	HUNGARY - HU

as follow-up to "**Financial Disclosure**<br><br>Enter a financial disclosure statement that describes the sources of funding for the work included in this submission. Review the submission guidelines for detailed requirements. View published research articles from *PLOS ONE* for specific examples.<br><br>This statement is required for submission and **will appear in the published article** if the submission is accepted. Please make sure it is accurate.<br><br>**Funded studies**<br>Enter a statement with the following details:<br>• Initials of the authors who received each award<br>• Grant numbers awarded to each author<br>• The full name of each funder<br>• URL of each funder website<br>• Did the sponsors or funders play any role in the study design, data collection and analysis, decision to publish, or preparation of the manuscript?<br><br>Did you receive funding for this work?"	
**Competing Interests**<br><br>Use the instructions below to enter a competing interest statement for this submission. On behalf of all authors, disclose any competing interests that could be perceived to bias this work—acknowledging all financial support and any other relevant financial or non-financial competing interests.<br><br>This statement is required for submission and **will appear in the published article** if the submission is accepted. Please make sure it is accurate and that any funding sources listed in your Funding Information later in the submission form are also declared in your Financial Disclosure statement.	The authors have declared that no competing interests exit.

View published research articles from *PLOS ONE* for specific examples.<br><br>**NO authors have competing interests**<br><br>Enter: *The authors have declared that no competing interests exist.*<br><br>**Authors with competing interests**<br><br>Enter competing interest details beginning with this statement:<br><br>*I have read the journal's policy and the authors of this manuscript have the following competing interests: [insert competing interests here]*<br><br>* typeset	
**Ethics Statement**<br><br>Enter an ethics statement for this submission. This statement is required if the study involved:<br><br>• Human participants<br>• Human specimens or tissue<br>• Vertebrate animals or cephalopods<br>• Vertebrate embryos or tissues<br>• Field research<br><br>Write "N/A" if the submission does not require an ethics statement.<br><br>General guidance is provided below. Consult the submission guidelines for detailed instructions. **Make sure that all information entered here is included in the Methods section of the manuscript.**	N/A

Powered by Editorial Manager® and ProduXion Manager® from Aries Systems Corporation

**Format for specific study types**<br><br>**Human Subject Research (involving human participants and/or tissue)**<br>• Give the name of the institutional review board or ethics committee that approved the study<br>• Include the approval number and/or a statement indicating approval of this research<br>• Indicate the form of consent obtained (written/oral) or the reason that consent was not obtained (e.g. the data were analyzed anonymously)<br><br>**Animal Research (involving vertebrate animals, embryos or tissues)**<br>• Provide the name of the Institutional Animal Care and Use Committee (IACUC) or other relevant ethics board that reviewed the study protocol, and indicate whether they approved this research or granted a formal waiver of ethical approval<br>• Include an approval number if one was obtained<br>• If the study involved *non-human primates*, add *additional details* about animal welfare and steps taken to ameliorate suffering<br>• If anesthesia, euthanasia, or any kind of animal sacrifice is part of the study, include briefly which substances and/or methods were applied<br><br>**Field Research**<br><br>Include the following details if this study involves the collection of plant, animal, or other materials from a natural setting:<br>• Field permit number<br>• Name of the institution or relevant body that granted permission	
**Data Availability**<br><br>Authors are required to make all data underlying the findings described fully available, without restriction, and from the time of publication. PLOS allows rare exceptions to address legal and ethical concerns. See the PLOS Data Policy and FAQ for detailed information.	Yes - all data are fully available without restriction

A Data Availability Statement describing where the data can be found is required at submission. Your answers to this question constitute the Data Availability Statement and **will be published in the article**, if accepted.<br><br>**Important:** Stating 'data available on request from the author' is not sufficient. If your data are only available upon request, select 'No' for the first question and explain your exceptional situation in the text box.<br><br>Do the authors confirm that all data underlying the findings described in their manuscript are fully available without restriction?	
**Describe where the data may be found in full sentences. If you are copying our sample text, replace any instances of XXX with the appropriate details.**<br><br>• If the data are **held or will be held in a public repository**, include URLs, accession numbers or DOIs. If this information will only be available after acceptance, indicate this by ticking the box below. For example: *All XXX files are available from the XXX database (accession number(s) XXX, XXX.).*<br>• If the data are all contained **within the manuscript and/or Supporting Information files**, enter the following: *All relevant data are within the manuscript and its Supporting Information files.*<br>• If neither of these applies but you are able to provide **details of access elsewhere**, with or without limitations, please do so. For example:<br><br>*Data cannot be shared publicly because of [XXX]. Data are available from the XXX Institutional Data Access / Ethics Committee (contact via XXX) for researchers who meet the criteria for access to confidential data.*<br><br>*The data underlying the results presented in the study are available from (include the name of the third party*	All (gyepkezeles-21-22-final.qmd, gyepkezeles_kiserlet_csigaadatok_2022vel_kiegeszitve.tab, terepi_adatlapok_osszesitoje_kimutatashoz_3pont0.tab) files are available from the https://adattar.unideb.hu/ Dataverse data repository database (accession number(s) https://doi.org/10.48428/ADATTAR/ULAHSC).

Powered by Editorial Manager® and ProduXion Manager® from Aries Systems Corporation

and contact information or URL).<br>• This text is appropriate if the data are owned by a third party and authors do not have permission to share the data.<br><br>* typeset	
Additional data availability information:	

Powered by Editorial Manager® and ProduXion Manager® from Aries Systems Corporation

Manuscript
Click here to access/download;Manuscript;Farkas_et_al_clean_copy.docx

The effect of mowing and mulching on snail communities: an experiment in wet meadows

Roland Farkas[1,2,3*], Miklós Bán[3], Gergő Oláh[3], György Dudás[1], Zoltán Barta[3]

[1]Bükk National Park Directorate, Eger, Hungary

[2]Juhász-Nagy Pál Doctoral School, University of Debrecen, Debrecen, Hungary

[3]HUN-REN-DE Behavioural Ecology Research Group, Department of Evolutionary Zoology and Human Biology, University of Debrecen, Debrecen, Hungary

- Corresponding author

E-mail: farkasro@yahoo.com

Abstract

The condition of wet meadows nowadays depends mainly on human activities; the biodiversity and productivity of these habitats can only be maintained through appropriate management methods. Mowing and grazing are well-known traditional methods, but a new method, regular mulching – the shredding of plant material and its deposition in the area - is becoming more widely practised. To better understand the impact of these methods we directly compared their effects on snails, an invertebrate taxon common in these habitats. We experimentally manipulated two wet meadows in Northern Hungary, Europe, and surveyed their snail communities immediately before and fourteen months after treatment. Our results showed that mowing had a detectable negative effect on the snail communities, whilst mulching did not alter their characteristics. Therefore, mulching may be a promising candidate for wet meadow habitats.

Keywords: Gastropoda, biodiversity, abundance, management, conservation

Introduction

Grasslands, making up more than 40% of lands worldwide, are home to many specialist and endemic species (Petermann and Buzhdygan, 2021). Moreover, the development of human culture was also closely linked to grasslands. Initially, it was ~~grasslands, initially being~~ used for nomadic animal husbandry and later for the rearing of livestock and growing forage (Squires et al. 2018). Thus, in addition to their outstanding ecological value, grasslands are also highly important economically.

The extent and condition of grasslands have changed over time since the agricultural revolution, driven primarily by human activities (Poschlod and WallisDeVries 2002). While a few activities, ~~few~~, such as deforestation, even create new grasslands, their extent has generally shrunk at an increasing rate with the intensification of agriculture and urbanisation. Furthermore, the emergence of new agricultural technologies has also greatly altered other characteristics of these habitats, such as landscape diversity and biodiversity (Fischer et al. 2008). As a result, the vast majority of historical grasslands have been transformed and cultivated by humans, mainly for rearing and grazing farm animals (Hejcman et al. 2013) and only small areas of pristine grassland remain. Many species historically endemic to grasslands were, however, able to settle in and survive on cultivated grasslands b~~efore their original habitats had completely disappeared.~~ Consequently, in modern times the fate of grassland communities strongly depends on their proper management by humans, which should be given a high priority.

The existence of wet meadows requires their soil to be saturated with water for at least part of the growing season. This wetness creates conditions which significantly isolates the habitat from its surroundings, offering unique conditions for species with specific habitat requirements. Because of these specific hydrological conditions, wet meadows are highly sensitive to environmental changes and are negatively affected by both current climatic changes and farming (Joyce et al. 2016). Consequently, wet meadows are an especially vulnerable form of grasslands, which have suffered significant reduction in area across Europe over the past few centuries (Zedler and Kercher, 2005). As a result, ~~the extent of wet meadows is decreasing in all EU countries and~~ they are classified as threatened by the European Red List of Habitats (Jansen et al. 2016). ~~Consequently, it is of paramount importance to investigate appropriate management methods of wet meadows.~~

repetition from lines 59-60

repetition from lines 50-51

In the absence of human intervention, natural succession processes usually take place on modern grasslands, resulting in spontaneous reforestation where the encroachment of shrubs and woody plants threatens the persistence of grassland communities and can also negatively impact farming activities (Moog et al. 2002). A striking example of these changes can be seen in Central and Eastern Europe, where the political and economic changes of the late 1980's and early 1990's drastically altered the agricultural landscape. Large-scale changes in ownership and economic restructuring led to the regional abandonment of large grassland areas, many of which are still in this state today (Kuemmerle et al. 2008; Halada et al. 2017). As grasslands harbour significant biodiversity and are important for farming (Petermann and Buzhdygan, 2021), the generally-accepted and desirable strategy is to maintain their current condition, rather than allowing them to return to an ancestral forested state.

Traditionally, the main methods of preventing spontaneous reforestation were grazing and mowing. More recently, the process of mechanised mulching has been developed to manage (Mašková et al. 2009) and recultivate abandoned grassland habitats (Liira et al. 2009). Unfortunately, mulching has several different meanings in English and ~~Unfortunately,~~ the specific mulching method is often not clearly defined in scientific articles (e.g. Pižl and Starý 2001; Babálová and Štrbová 2014)~~, despite the fact that mulching has several different meanings in English.~~ This methodological ambiguity makes it difficult to understand the effects of mulching on biodiversity. For instance, one common definition of mulching is the process of spreading plant material harvested elsewhere, for example to protect economic crops (e.g. Bautista 2018). Another is when plant material cut (but not shredded) by a mower machine is left to decompose on the spot (e.g. Liira et al. 2009, Klimeš et al. 2013). Here, we use a third definition, whereby the standing vegetation on the meadow is shredded with a flail mulcher machine, with the resulting material spread evenly over the surface of the meadow (i.e. not removed). This definition is also used by Moog et al. (2002), Schmitt (2003), Doležal et al. (2011), Pavlů et al. (2016), Gaisler et al. (2019), and Georgi et

Regular mulching is commonly used for habitat restoration in nature conservation areas to remove shrubs and smaller trees from abandoned grasslands.

al. (2022, 2023). ~~Regular mulching can be used to remove shrubs and smaller trees from abandoned grasslands and restore them within a few years. It is commonly used for habitat restoration in nature conservation areas.~~ One of the reasons for the spread of mulching is that it is more economical than traditional methods (Liira et al. 2009)~~. Mulching requires~~ as it requires only a one-time intervention, while cutting, drying, raking, baling and transporting the bales during traditional mowing require more investment.

Mowing and mulching can have various effects on the environment and its plant and animal communities. Direct effects from the moving parts of the machines cause damage to both the flora and fauna (Humbert et al. 2010, Steidle et al. 2022). Indirect effects arise from the associated changes to the physical and chemical properties of the habitat (Humbert et al. 2009). In this case, it is important whether the plant material is removed from the area or left to decompose in place. When vegetation is removed, direct irradiance can penetrate deeper, even to the surface of the soil. This can result in higher temperatures and hence stronger evaporation compared to similar habitats with closed vegetation (Gardiner and Hassal 2009, Hesslerová et al. 2013). On the other hand, covering the surface with cut plant material can result in lower temperatures (Dudas et al. 2016) and higher soil humidity (Shirish et al. 2013). Mowing and mulching also changes the soil microbiome, influencing decomposition processes and the amount of available nutrients (Kaštovská et al. 2005, Pavlů et al. 2016, Horan et al. 2018). Biomass accumulated on the soil surface significantly increases the activity of decomposing organisms and enzymes in the soil (Jabran 2022).

The impact of management on grasslands depends greatly on the method used and the characteristics of the habitat concerned, and is an important research topic. Most work so far has looked at how management practices change plant communities, in particular their species composition, biomass or priority species groups. Plant communities in mowed areas have

generally higher diversity than those in unmanaged, abandoned areas, except when the area is intensively managed. This has especially been demonstrated in dry, semi-arid, nutrient-poor meadows and mountain pastures (Ryser et al. 1995). In these areas, both mowing and mulching have generally been found to have a positive impact on maintaining species-rich habitats (Moog et al. 2002, Gaisler et al. 2004, Klimeš et al. 2013, Doležal et al. 2011). Limited evidence suggests that both mowing and mulching increase species richness in wet, alluvial habitats (Liira et al. 2009).

repetition from lines 113-115

In contrast to our more detailed knowledge on the impact of mowing and mulching on flora, their effects on fauna, especially on invertebrates, has been less well studied. According to the sparse literature, mowing mostly has a negative impact on the abundance and diversity of some invertebrate groups (snails: Pech et al. 2015, Farkas et al. 2024, less mobile spiders: Cattin et al. 2003, dung beetles: Frank et al. 2017, orthopterans: Chisté et al. 2016). Mulching has also been shown to have a similar negative effect on several groups' abundance and diversity (butterflies: Babálová and Štrbová 2014, bees and wasps: Georgi et al. 2022, insect larvae, flower visitors: Georgi et al. 2023). However, the impact of these management methods can be influenced by a number of factors, such as the timing and the frequency of treatments (Humbert et al. 2009, Georgi et al. 2023). The literature suggests that, independently of habitat type, it is difficult to draw general conclusions about the effects of grassland management on animals because different taxa living in the same habitat have been observed to respond differently to the same treatment (Pižl and Starý 2001, Cizek et al. 2012). Consequently, we need more detailed studies targeted at specific groups to better understand the effects of grassland management practices.

Snails (*Gastropoda*) are common and widespread inhabitants of wet meadows (Welter-Schultes 2012). They are significant actors in the decomposition processes (Newell 1967): they stimulate the activity of microbial detritivores by fragmenting organic materials, and

their excretion of faeces and mucus production provides favourable conditions for microbial life (Theenhaus and Scheu, 1996). Furthermore, the high water content of their bodies, their slow movement and limited dispersal ability make snail populations especially sensitive to environmental change. This makes them excellent subjects for studying the effects of environmental factors in general, and impacts of management practices in particular (Pech et al. 2015, Wehner et al. 2019). Nevertheless, the impact of mulching on snail communities is unexplored.

The aim of our study was to identify sustainable management methods for wet meadows by using snails as a target taxon group~~. The locations of our study were~~ on wet meadows that had remained untouched for decades. We mowed and mulched portions of these areas using standard agricultural machinery, following a stratified experimental design. After fourteen months, we returned to collect data on the current state of the snail communities and investigated the effects of each treatment on their abundance and diversity.

Mowing removes biomass, while mulching leaves it in place, so we expected different effects from the two treatments. Both treatments are drastic interventions in habitats that have been abandoned for decades, so we also expected to find significant differences compared to the control areas. We chose the fourteen-months interval to investigate the effects of treatments because early recognition of adverse impacts is important to prevent valuable habitats from being treated with harmful methods in the longer term.

Methods

Study area

The experiment was carried out at two sites in Northern Hungary, Europe (**Fig 1**). The central WGS84 coordinates of the sites are the following: Szilvásvárad X= 48.1250, Y= 20.3920;

Tarnalelesz X= 48.0908, Y= 20.1506. Both sites are located in stream valleys of a hilly area, west of the Bükk Mountains. This area is characterised by high forest cover, with wet meadows occurring almost exclusively along the valley bottom watercourses. Annual rainfall in this area ranges between 600-700 mm and the average annual temperature is around 9°C (Dövényi 2010). In the valley bottoms meadow moulding soils have developed on clayey, loamy, sandstone bedrock (Dövényi 2010). In both sites, the habitats studied were non-tussock tall-sedge beds, with a dominance of sedge (*Carex acutiformis*). The characteristic associated species observed were *Lysimachia vulgaris*, *Lythrum salicaria*, *Cirsium oleraceum* and *Urtica dioica*. Both sites were flat, horizontal areas with an apparently uniform structure. No bushes or trees were present in any of the sites. An important criterion for the selection of the sites was that they represent wet meadows that have not been farmed for the last 30 years. To verify this, a series of archive aerial photographs (www.fentrol.hu) were used.

Figure 1. The study sites.

Legend: Top centre: a map of Hungary with a grey circle indicating the location of the study areas. WGS 84 geographic coordinates are marked on the axes. Middle: photos of the habitats before the management. Bottom: detailed sampling design. Columns represent treatment plots, different colours indicate the type of treatment: white – control, light grey – mowed, dark grey – mulched. Randomly placed squares symbolize sampling points: orange - pretreatment, green – posttreatment.

Experimental design

A sample area of ~~45x15~~ 45 x 15 meters was marked out in each site. These were divided into 9 plots of 5 x 15 meters each. Both the edges of the sample area and the boundaries of the plots were marked with permanent stakes. Out of the 9 plots, 3 were mowed, 3 were mulched, and 3

Regular mulching is commonly used for habitat restoration in nature conservation areas to remove shrubs and smaller trees from abandoned grasslands.

remained untouched as controls. Treatments were assigned to the plots in a random order. Each plot was divided into 5 sections of 5x3 meters. In each of these, a sample was taken from a randomly selected point, resulting in 45 sampling points at each site (15 per treatment) (**Fig 1**). This gives n=30 replicates per treatment for the whole study (2 study sites x 3 treatment plots per site x 5 samples per plot). On June 28, 2021. (Szilvásvárad) and June 29, 2021. (Tarnalelesz), a malacological sampling took place at each sampling point before the start of management. Treatments were carried out a few days after this sampling, on July 1, 2021 at both sites. This is a typical time for farmers in the region to carry out farming activities on meadows. In the mowed plots, we cut the vegetation with a disc mower machine pulled by a tractor. The cut material was immediately removed from the surface using hand rakes. Mulching treatment was done by a flail mulcher machine pulled by a tractor to shred the vegetation into pieces smaller than 10 cm. The resulting material was not removed and evenly covered the surface. Both sites were then left undisturbed. Subsequently, on August 15, 2022 (Szilvásvárad) and August 14, 2022 (Tarnalelesz), we went back to the sites and again collected a malacological sample from each sampling point. Malacological sampling was carried out in the morning hours each time. Sampling dates were selected to ensure that the weather conditions were similar and rain-free during the two-day periods.

One malacological sample was taken per sampling point on each occasion. Sampling was performed by collecting the leaf litter and the top 1 cm thick layer of the soil from a ~~25X25~~ 25 x 25 cm quadrat. The collected samples were stored in airtight plastic bags in a cool, dark place. On the second day after collection, samples were floated: they were poured individually into buckets containing a volume of water significantly greater than the sample volume and left for half an hour. The floating material was then washed thoroughly in the buckets to remove any

trapped snail shells and discarded. The material remaining in the bucket, including the snails, was washed through a sieve with a mesh size of 0.75 mm. The hole size was chosen so as not to allow the passage of the smallest adult snail specimens. The material remaining on the sieve contained the washed snail shells and other small organic and inorganic debris. This was immediately placed in 70% ethanol and stored. Later, the snail shells were removed from these samples using a 3.5X magnifying glass. The sorted snail shells were identified to the species level using a stereo microscope (Motic SMZ-168 series, 10X magnification). All determinations were carried out by RF, and they were based on the works of Kerney et al. (1983), Glöer and Meier-Brook (2003) and Welter-Schultes (2012). Only fully developed and intact (not broken or incomplete) shells of live adults were counted in the study. Live specimens were considered to be those in which the body of the snail could be observed inside the translucent shell or by looking into the aperture of the specimens. Slugs were not included in the study because this sampling method is not suitable for their quantitative analysis (Cameron and Pokryszko, 2005).

Statistical analysis

All statistical analyses were performed in the R statistical environment (v4.2.2, R Core Team 2022). First, we took a community based approach by calculating the Bray-Curtis distance matrix between the sampling points by the vegdist function of the vegan package (ver. 2.6-8, Oksanen et al., 2024) and then partitioned the variance of the distance matrix by performing a PERMANOVA (McArdle and Anderson, 2001) with sampling time (pre- and posttreatment), treatment (control, mowing and mulching) and their interaction as explanatory terms (adonis2 function of vegan). If the different treatments differently affect the changes in snail communities over time one would expect a significant interaction between the variables of

treatment and sampling time. PERMANOVA performs random permutations of the data to derive the distributions of estimated parameters. In our model, spatial non-independence (i.e. data come from two separate areas) was controlled for by restricting permutations to within areas only (Bakker 2024).

Snail communities were further characterised by three community characteristics: (i) the total number of specimens, (ii) the number of species and (iii) Pielou's species evenness, H' = $\Sigma p_i \ln p_i / \ln S$, where p_i is the relative abundance of species i in the sample and S is the number of species in the sample (Pielou 1975). We fitted three generalised linear mixed effects models (GLMMs) to our data, one for each community characteristic. We entered treatment, sampling time and their interaction as fixed effects. We also took into account the statistical non-independence caused by spatial proximity by entering random effects into the models. Random effects were entered as sampling point ID nested within plot ID nested within site ID. Note, that we had to remove the random effect terms from the model for evenness, because this model did not converge as the variance explained by the random effect terms was essentially zero. We used a negative binomial error distribution for the number of specimens, and normal error distributions for the number of species and evenness. Inspecting the model fits with the DHARMa R package (v0.4.6, Hartig 2022) did not indicate any deviations from model assumptions.

We also analysed how treatments affected the number of individuals for the three most common species in the samples (*Carychium minimum*, *Vertigo angustior* and *Vallonia pulchella*). We fitted separate GLMM models for each species data with the same fixed and random effects as specified above. We used a negative binomial error distribution for all three species. Model assumptions were also checked by the DHARMa package.

All GLMM models were fitted with the 'glmmTMB' function of the glmmTMB package (v1.1.7, Brooks et al. 2017). If a significant interaction was detected (by log ratio tests) we

tested posthoc which treatment groups were significantly different from each other by the emmeans function of the emmeans package (v1.8.4-1, Lenth 2023).

Results

During the study, 5233 living individuals of 20 species were found in the two sample sites combined (Fig. 2)~~. We show the total number of individuals by species is shown in **Fig2**~~. Total number of individuals in different study sites, treatment plots and sampling times is shown in **Appendix 1**.

Figure 2. Total number of living individuals in the samples.

Numbers of individuals are plotted on a logarithmic scale. Species are shown with decreasing numbers of individuals from top to bottom.

PERMANOVA analysis indicated a significant interaction between sampling time and treatment ($F_{2,169}$ = 2.209, p = 0.003, R^2 = 0.023) suggesting that communities changed differently with different treatments. Investigating the fitted parameters of the PERMANOVA model revealed (i) no initial differences between the communities of different treatments ($F_{2,169}$ = 1.033, p = 0.251, R^2 = 0.010), (ii) significant changes with sampling time ($F_{1,169}$ = 17.619, p = 0.001, R^2 = 0.091) and that (iii) only the mowed community changed differently from the control ($F_{1,169}$ = 2.921, p = 0.006, R^2 = 0.015).

Analysing community characteristics (abundance, species richness and evenness) with GLMMs, there were no significant differences between the mowed, mulched and control plots before treatment in any characteristic (**Appendix 2**). However, we found a significant

interaction between treatment and sampling time for all community characteristics: number of living individuals (χ_2^2 = 6.99, p = 0.03), species number (χ_2^2 = 11.32, p = 0.01) and evenness (χ_2^2 = 6.26, p = 0.04). This indicates that different treatments affected the snail communities differently.

Next, by comparing treatment groups, we examined how snail populations changed due to the treatments (**Fig 3, Appendix 3**). The estimated number of living individuals decreased in all three management types, but this decrease was only significant in the mowed areas, indicating that the mowing itself likely caused a decrease. In terms of species numbers, again only mowing caused a significant decrease. For evenness, we also found a significant difference only in the mowed areas, but in this case the evenness increased, meaning that the abundance of each species in the community became more similar.

Figure 3. The impact of treatment on three community characteristics

The impact of treatment a) on the number of living individuals, b) on the number of species and c) on evenness. Vertical error bars indicate standard errors of the estimates derived by the emmeans function from the fitted GLMM models (***: p<0.001, *: p<0.05, ns: p>0.05).

Finally, in addition to examining entire communities, we investigated whether treatments affected the abundance of the three most common species (*Carychium minimum*, *Vallonia pulchella*, *Vertigo angustior*). In C. ~~*Carychium*~~ *minimum* the abundance decreased significantly with sampling time (χ^2_1 = 97.16, p < 0.001) independently of treatment (effect of treatment: χ^2_2 = 0.72, p = 0.696; interaction: χ^2_2 = 2.85, p = 0.241). In the case of V. ~~*Vallonia*~~ *pulchella* neither treatments (χ^2_2 = 3.55, p = 0.169), sampling times (χ^2_1 = 2.62, p = 0.105), nor their

interaction ($\chi^2_2 = 3.98$, p = 0.137) significantly affected abundance. Only for ~~*Vertigo*~~ Ve. *angustior* did treatments (interaction: $\chi^2_2 = 7.90$, p = 0.019) differently affect the abundance; mulching significantly increased the number of individuals (posthoc test: z = -3.25, p = 0.001) while the other two treatments did not have such ~~any~~ effect (control, posthoc test: z = -1.53, p = 0.127; mowing, posthoc test: z = -0.81, p = 0.417; **Fig 4**).

Figure 4. The impact of treatment on the number of living *Vertigo angustior* individuals. Vertical error bars indicate standard errors of the estimates (**: p<0.01, *: p<0.05, ns: p>0.05).

Discussion

We found that snail communities declined significantly in mowed ~~areas~~, but not mulched ~~ones~~ areas, indicating that mowing can have a detectable negative impact on snail communities. This seemingly contradicts our finding that Pielou’s evenness increased on mowed areas. However, the ~~two~~ results are not necessarily mutually exclusive, since under certain conditions, diversity indices can increase despite a decrease in number of individuals and species. For instance, if the decrease is larger in more abundant species the distribution of individuals among species becomes more uniform and evenness increases. In cases like this, we must define our optimal outcome, and it seems sensible to choose a smaller decrease in abundance as more optimal than an increase in evenness. So in this situation, our finding remains, that mowing has negative effects on snail communities whereas mulching does not.

There has been relatively little research on the effects of mowing on snails, and, as far as we know, there is no previous work regarding the effects of mulching. Previous research has mainly focused on longer-term effects over several years. For instance, Pech and colleagues

(2015; working in oligotrophic grasslands), and Farkas and colleagues (2024; on valley-bottom wet meadows) found long-term reductions in abundance and species numbers due to mowing, but Wehner and colleagues (2019; over a wide range of meadows) found an increase in snail community diversity in mowed areas, while no difference in abundance was found. All three studies are similar in that they were conducted on areas that were previously mowed (albeit for varying durations), while our current study was conducted in wet meadows that were previously untreated for decades. In addition, the above studies investigated the effects of mowing not by comparing samples taken before and after treatment on the same site, but by analyzing data from mowed and adjacent unmowed areas. Despite these methodological differences, our result on the detrimental effects of mowing is similar to two of these previous studies.

Our findings pertain to snail communities, yet wet meadows harbor numerous other abundant invertebrate taxa, which may respond differently to different management practices. Mowing had negative effects on abundance of less mobile spiders (Cattin et al. 2003), species richness (Cizek et al. 2012) and diversity (Chisté et al. 2016) of orthopterans, and biomass of dung beetles (Frank et al. 2017). Other studies have found that mulching decreased abundance of herbivorous insect larvae, flower visitors (Georgi et al. 2023) and herbivore-hunting wasp (Georgi et al. 2022) communities. Pizl and Stary (2001) conclude that abundance of earthworms is lower on mowed than mulched sites, Schmitt (2003) found that the abundance of butterflies is higher in mowed than mulched plots. However, the number of studied invertebrate groups is limited. This makes it difficult to draw general conclusions about the effects of treatments on entire invertebrate communities in a particular habitat. Moreover, the range of habitats studied is broad, with varying initial conditions: some studies monitor the effects of different ongoing treatments (Schmitt 2003, Chisté et al. 2016, Frank et al. 2017),

while others established new experimental setups on habitats that had earlier undergone some form of regular treatment (Georgi et al. 2022, 2023). However, there are no previous studies where the initial state of the habitat, like ours, was untreated for decades.

Our results indicate that members of snail communities did not respond uniformly to the management. Of the three most common species we examined, both *Carychium minimum* and *Vertigo angustior* are limited to wetland habitats, but only the latter was affected by the management. *Vallonia pulchella* has a wider range of habitat preferences, and was also unaffected by the treatment. Similar results were observed in management studies with other taxa. Cizek and colleagues (2012) found that the abundance of different ground beetle species changed differently with the same treatment. Pižl and Starý (2001) showed differential changes in abundance for different species of oribatids living above the soil surface in response to management. Both sources agree that generalist species tolerate changes better than specialists. *Vertigo angustior* is still considered a common species in Hungary, but its distribution in Western Europe has drastically declined, which can be attributed to changing and increasingly intensive agriculture (European Environment Agency 2013). Therefore, the appropriate choice of management practices is of paramount importance for its preservation. The observed consistent declines in abundance of snail communities at both sites for all treatments were likely due to historic drought and heatwaves ~~occurred~~ occurring in Hungary in 2022 (Szentes 2023). In addition to these changes, management methods can have both direct (e.g. caused by the machines themselves) and indirect effects (e.g. caused by habitat transformation) on invertebrate communities. In our case, the changes observed in the mowed snail community are most probably due to indirect effects. The majority of the snail species studied live in the litter layer of the habitat, and the rotating parts of the equipment do not affect this layer. ~~Trampling~~ Being driven over by tractor wheels also has a direct negative effect on species

living on the ground (Humbert et al. 2010) but in our study the mowed and mulched plots were driven over ~~trampled~~ to the same extent. Instead, the significantly higher decrease in abundance from mowing might have been mainly due to effects such as the sudden increase in irradiance and temperature, and a decrease in humidity (Shirish et al. 2013, Dudas et al. 2016).

In our study, mowing was defined as cutting the vegetation and immediately removing the biomass, which is different to real-life agricultural practices. ~~In practice, having cut it,~~ farmers typically leave the biomass remains on the surface for days, while it is dried, turned, windrowed, and finally baled and removed. Leaving the cut hay temporarily out might prevent desiccation, thus reducing the negative effects of mowing. However, in nature conservation treatments focused on small areas, the immediate removal of the cut biomass is standard practice. Therefore, our study rather ~~better~~ represents current nature conservation, ~~rather~~ than agricultural~~,~~ practices.

Careful selection of management conditions can significantly reduce their negative impact on flora and fauna. These include staggering treatments throughout the year or leaving parts of the habitat unmanaged (Pech et al. 2005, Cizek et al. 2012). The negative effects of treatments could also be prevented or reduced by tailoring them to the life cycle of invertebrates (Georgi et al. 2022, 2023). It may also be beneficial not to manage an area every year, <u>but this can threaten the survival of the whole habitat beyond a certain reduction in intensity</u> (Ryser et al. 1995, Moog et al. 2002). unclear

Our experimental study had promising results, but we are still far from having sufficient knowledge of the effects of grassland treatments. Our research was limited to one season, two sites and a comparison of three treatment methods. Short-term results are important because they can guide future treatments. On the other hand, to achieve sustainability, longer-term studies are needed~~. Our current knowledge in this area is still limited,~~ and it would be useful to expand our study in ~~in~~ multiple ways. First, by including additional litter or soil-dwelling taxa, in order

to get a more complete idea of the effects of each treatment on entire ecosystems. Second, by comparing both mowing and mulching as well as their common combinations, since in practice, management methods are often applied together, resulting in potentially different effects than just one treatment alone. Third, including different spatiotemporal patterns and intensities of treatments would allow us to even better understand the effects of real-world agricultural practices.

To summarise, significantly more research is needed to properly understand the effects of grassland management on flora and fauna. This would allow us to optimise real-world agricultural practices to each habitat, ensuring the most suitable management practices are used and thus best preserving biodiversity.

Conclusions

~~Wet meadows have been managed and cultivated by humans since ancient times. New methods and increasingly intensive agricultural practices are significantly transforming these valuable habitats. While the impact of treatments on vegetation is somewhat understood, little is known about the fate of animal communities, especially invertebrate ones.~~ Through a controlled grassland management experiment, we examined the effects of mowing and mulching on the snail communities of wet meadows. Our results indicate ~~that significant changes can be observed fourteen months after treatment. The negative impact of mowing was evident within this time frame, while mulching did not lead to significant changes. Mulching thus seems to be~~ that mulching is a suitable way for short-term interventions of grassland areas without the negative effects of mowing on snail communities. ~~Sustaining grasslands in the long term requires not only maintaining their habitat structure but also preserving their quality and biodiversity. Given the observed negative effects of mowing, selecting appropriate methods is crucial. Within a given area, numerous taxa are present, each responding~~

~~differently to interventions. Hence, conducting similar studies across diverse animal groups is necessary. In addition, whilst~~ Hwever, while data on the effects of different management methods after one year are useful, there is still a lack of long-term monitoring of grassland management, particularly regarding mulching on wet meadows. Initiating, supporting and maintaining more long-term research projects is crucial. In addition to economic considerations, ecological aspects of meadow management must also be taken into account to ensure sustainability for the future.

Acknowledgement

We are grateful to Tamás Székely, Jr. for his comments on the manuscript. The study was prepared with the professional support of the Doctoral Student Scholarship Program of the Co-operative Doctoral Program of the Ministry of Innovation and Technology financed from the National Research, Development and Innovation Fund (Hungary). The study was supported by a National Research, Development and Innovation Office grant (NKFIH K138503 to ZB). ZB was supported by a National Research, Development and Innovation Fund grant (TKP2021-NKTA-32).

Literature

Babálová, M., Štrbová, E., 2014. Influence of grasslands management on diversity of the diurnal butterflies of Vlkolínec Village (Central Slovakia). Ecology and noospherology 83–91.

Bakker, Jonathan D. 2024. Applied Multivariate Statistics in R. University of Washington. https://uw.pressbooks.pub/appliedmultivariatestatistics/.

Bautista, F., Delgado, M., Estrada-Medina, H., 2008. Effect of legume mulches and cover crops on earthworms and snails. Tropical and subtropical agroecosystems 8, 45–60.

Brooks, M. E., Kristensen, K., van Benthem, K. J., Magnusson, A., Berg, C. W., Nielsen, A., Skaug, H. J., Maechler M., Bolker, B. M. 2017. glmmTMB Balances Speed and Flexibility Among Packages for Zero-inflated Generalized Linear Mixed Modeling. The R Journal, 9(2), 378-400. doi: 10.32614/RJ-2017-066.

Cattin Blandenier, M.-F., Banašek-Richter, C., Bersier, L-F., 2003. The impact of mowing as a management strategy for wet meadows on spider (Araneae) communities. Biological Conservation 113, 179–188. https://doi.org/10.1016/S0006-3207(02)00297-5

Chisté, M. N., Mody, K., Gossner, M. M., Simons, N. K., Köhler, G., Weisser, W. W., Blüthgen, N., 2016. Losers, winners, and opportunists: How grassland land-use intensity affects orthopteran communities. Ecosphere 7, e01545. https://doi.org/10.1002/ecs2.1545

Cizek, O., Zámečník, J., Tropek, R., Kocarek, P., Konvicka, M., 2012. Diversification of mowing regime increases arthropods diversity in species-poor cultural hay meadows. J Insect Conserv 16, 215–226. https://doi.org/10.1007/s10841-011-9407-6

Doležal, J., Mašková, Z., Lepš, J., Steinbachová, D., de Bello, F., Klimešová, J., Tackenberg, O., Zemek, F., Květ, J., 2011. Positive long-term effect of mulching on species and functional trait diversity in a nutrient-poor mountain meadow in Central Europe. Agriculture, Ecosystems & Environment, Understanding species and community response to environmental change - a functional trait perspective 145, 10–28. https://doi.org/10.1016/j.agee.2011.01.010

Dövényi, Z. (ed.), 2010. Magyarország kistájainak katasztere. MTA Földrajzi Kutatóintézet, Budapest.

Dudás, P., Menyhárt, L., Gedeon, C., Ambrus, G., Tóth, F., 2016. The effect of hay mulching on soil temperature and the abundance and diversity of soil-dwelling arthropods in potato fields. Eur. J. Entomol. 113, 456–461. https://doi.org/10.14411/eje.2016.059

European Environmental Agency, 2013. Vertigo angustior - Report under the Article 17 of the Habitats Directive Period 2007-2012. https://projects.eionet.europa.eu/habitat-art17report/library/2007-2012-reporting/factsheets/species/molluscs/vertigo-angustior/download/en/1/vertigo-angustior.pdf

Farkas, R., Bán, M., Barta, Z., 2024. Mowing wet meadows reduces the health of their snail communities. PeerJ 12:e16783 DOI 10.7717/peerj.16783

Frank, K., Hülsmann, M., Assmann, T., Schmitt, T., Blüthgen, N., 2017. Land use affects dung beetle communities and their ecosystem service in forests and grasslands. Agriculture, Ecosystems & Environment 243, 114–122. https://doi.org/10.1016/j.agee.2017.04.010

Gaisler, J., Hejcman, M., Pavlů, V., 2004. Effect of different mulching and cutting regimes on the vegetation of upland meadow. Plant Soil Environ. 50, 324–331. https://doi.org/10.17221/4039-PSE

Gaisler, J., Pavlů, L., Nwaogu, C., Pavlů, K., Hejcman, M., Pavlů, V. V., 2019. Long-term effects of mulching, traditional cutting and no management on plant species composition of improved upland grassland in the Czech Republic. Grass and Forage Science 74, 463–475. https://doi.org/10.1111/gfs.12408

Gardiner, T., Hassall, M., 2009. Does microclimate affect grasshopper populations after cutting of hay in improved grassland? J Insect Conserv 13, 97–102. https://doi.org/10.1007/s10841-007-9129-y

Georgi, M. M., Fornoff, F., Gärtner, S. M., Neitzel, S., Geist, A., Klein, A-M., 2022. Timing and mulching frequency affected the number of nests of cavity-nesting wasps that hunt for aphids in forest meadows. J Insect Conserv 26, 973–981. https://doi.org/10.1007/s10841-022-00442-y

Georgi, M. M., Gärtner, S. M., Förschler, M. I., Buse, J., Fornoff, F., Ssymank, A., Oelmann, Y., Klein, A-M., 2023. Mulching time of forest meadows influences insect diversity. Insect Conservation and Diversity 16, 368–378. https://doi.org/10.1111/icad.12629

Glöer, P., Meier-Brook, C., 2003. Süsswassermollusken: Ein Bestimmungsschlüssel für die Bundesrepublik Deutschland. Deutscher Jugendbund für Naturbeobachtung, Göttingen.

Fischer, M., Rudmann-Maurer, K., Weyand, A., Stöcklin, J., 2008. Agricultural Land Use and Biodiversity in the Alps: How Cultural Tradition and Socioeconomically Motivated Changes Are Shaping Grassland Biodiversity in the Swiss Alps. Mountain Research and Development 28, 148–155.

Halada, Ľ., David, S., Hreško, J., Klimantová, A., Bača, A., Rusňák, T., Buraľ, M., Vadel, Ľ., 2017. Changes in grassland management and plant diversity in a marginal region of the Carpathian Mts. in 1999–2015. Science of The Total Environment 609, 896–905. https://doi.org/10.1016/j.scitotenv.2017.07.066

Hartig F., 2022. DHARMa: Residual Diagnostics for Hierarchical (Multi-Level / Mixed) Regression Models_. R package version 0.4.6, <https://CRAN.R-project.org/package=DHARMa>.

Hejcman, M., Hejcmanová, P., Pavlů, V., Beneš, J., 2013. Origin and history of grasslands in Central Europe – a review. Grass and Forage Science 68, 345–363. https://doi.org/10.1111/gfs.12066

Hesslerová, P., Pokorný, J., Brom, J., 2013. Daily dynamics of radiation surface temperature of different land cover types in a temperate cultural landscape: Consequences for the local climate. Ecological Engineering 54, 145–154. https://doi.org/10.1016/j.ecoleng.2013.01.036

Horan, B., Hennessy, D., O'Donovan, M. (Eds.), 2018. Sustainable meat and milk production from grasslands: proceedings of the 27th General Meeting of the European Grassland Federation,

Cork, Ireland, 17 - 21 June 2018, Grassland Science in Europe$Ivolume 23. Presented at the European Grassland Federation, Wageningen Academic Publishers, Wageningen.

Humbert, J-Y., Ghazoul, J., Sauter, G., Walter, T., 2010. Impact of different meadow mowing techniques on field invertebrates. Journal of Applied Entomology 134, 592–599. https://doi.org/10.1111/j.1439-0418.2009.01503.x

Humbert, J-Y., Ghazoul, J., Walter, T., 2009. Meadow harvesting techniques and their impacts on field fauna. Agriculture, Ecosystems & Environment 130, 1–8. https://doi.org/10.1016/j.agee.2008.11.014

Jabran, K., 2019. Role of Mulching in Pest Management and Agricultural Sustainability, SpringerBriefs in Plant Science. Springer International Publishing, Cham. https://doi.org/10.1007/978-3-030-22301-4

Janssen, J., Rodwell, J., García Criado, M., Gubbay, S., Haynes, T., Nieto, A., Sanders, N., Landucci, F., Loidi, J., Ssymank, A., Tahvanainen, T., Valderrabano, M., Acosta, A., Aronsson, M., Arts, G., Attorre, F., Bergmeier, E., Bijlsma, R-J., Bioret, F., Biţă-Nicolae, C., Biurrun, I., Calix, M., Capelo, J., Čarni, A., Chytrý, M., Dengler, J., Dimopoulos, P., Essl, F., Gardfjell, H., Gigante, D., Giusso del Galdo, G., Hájek, M., Jansen, J., Kapfer, J., Mickolajczak, A., Molina, J.A., Molnár, Z., Paternoster, D., Piernik, A., Poulin, B., Renaux, B., Schaminée, J. H. J., Šumberová, K., Toivonen, H., Tonteri, T., Tsiripidis, I., Tzonev, R., Valachovič, M., 2016. European Red List of Habitats Part 2. Terrestrial and freshwater habitats. Publications Office of the European Union, Luxembourg. https://doi.org/10.2779/091372

Joyce, C. B., Simpson, M., Casanova, M., 2016. Future wet grasslands: ecological implications of climate change. Ecosystem Health and Sustainability 2, e01240. https://doi.org/10.1002/ehs2.1240

Kaštovská, E., Simek, M., Santruckova, H., 2005. Microbial transformation of organic matter in soils of montane grasslands under different management. Applied Soil Ecology 28, 225–235. https://doi.org/10.1016/j.apsoil.2004.08.002

Kerney, M. P., Cameron, R. A. D., Jungbluth, J. H., 1983. Die Landschnecken Nord- und Mitteleuropas: ein Bestimmungsbuch für Biologen und Naturfreunde. Paul Parey, Hamburg und Berlin.

Klimeš, L., Hájek, M., Mudrák, O., Dančák, M., Preislerová, Z., Hájková, P., Jongepierová, I., Klimešová, J., 2013. Effects of changes in management on resistance and resilience in three grassland communities. Applied Vegetation Science 16, 640–649. https://doi.org/10.1111/avsc.12032

Kuemmerle, T., Hostert, P., Radeloff, V. C., van der Linden, S., Perzanowski, K., Kruhlov, I., 2008. Cross-border Comparison of Post-socialist Farmland Abandonment in the Carpathians. Ecosystems 11, 614–628. https://doi.org/10.1007/s10021-008-9146-z

Lenth, R. (2023). _emmeans: Estimated Marginal Means, aka Least-Squares Means_. R package version 1.8.4-1, <https://CRAN.R-project.org/package=emmeans>.

Liira, J., Issak, M., Jõgar, Ü., Mändoja, M., Zobel, M., 2009. Restoration Management of a Floodplain Meadow and Its Cost-Effectiveness — the Results of a 6-Year Experiment. Annales Botanici Fennici 46, 397–408. https://doi.org/10.5735/085.046.0504

Mašková, Z., Doležal, J., Květ, J., Zemek, F., 2009. Long-term functioning of a species-rich mountain meadow under different management regimes. Agriculture, Ecosystems & Environment 132, 192–202. https://doi.org/10.1016/j.agee.2009.04.002

McArdle, B. H., Anderson, M. J., 2001. Fitting Multivariate Models to Community Data: A Comment on Distance-Based Redundancy Analysis. Ecology 82, 290–297. https://doi.org/10.2307/2680104

Moog, D., Poschlod, P., Kahmen, S., Schreiber, K-F., 2002. Comparison of species composition between different grassland management treatments after 25 years. Applied Vegetation Science 5, 99–106. https://doi.org/10.1111/j.1654-109X.2002.tb00539.x

Newell, P. F. 1967. Mollusca. In Burgess A, Raw F. (ed) Soil Biology. Academic Press, London, pp. 413-444.

Oksanen J., Simpson G., Blanchet F., Kindt R., Legendre P., Minchin P., O'Hara R., Solymos P., Stevens M., Szoecs E., Wagner H., Barbour M., Bedward M., Bolker B., Borcard D., Carvalho G., Chirico M., De Caceres M., Durand S., Evangelista H., FitzJohn R., Friendly M., Furneaux B., Hannigan G., Hill M., Lahti L., McGlinn D., Ouellette M., Ribeiro Cunha E., Smith T., Stier A., Ter Braak C., Weedon J. 2024. _vegan: Community Ecology Package_. R package version 2.6-8, <https://CRAN.R-project.org/package=vegan>.

Pavlů, L., Gaisler, J., Hejcman, M., Pavlů, V. V., 2016. What is the effect of long-term mulching and traditional cutting regimes on soil and biomass chemical properties, species richness and herbage production in Dactylis glomerata grassland? Agriculture, Ecosystems & Environment 217, 13–21. https://doi.org/10.1016/j.agee.2015.10.026

Pech, P., Dolanský, J., Hrdlička, R., Leps, J., 2015. Differential response of communities of plants, snails, ants and spiders to long-term mowing in a small-scale experiment. Community Ecology 16, 115–124. https://doi.org/10.1556/168.2015.16.1.13

Petermann, J. S., Buzhdygan, O. Y., 2021. Grassland biodiversity. Current Biology 31, R1195–R1201. https://doi.org/10.1016/j.cub.2021.06.060

Pielou, E. C. 1975. Ecological diversity. John Wiley & Sons, New York.

Pižl, V., Starý, J., 2001. The effects of mountain meadows management on soil fauna communities (on example of earthworms and oribatid mites). Silva Gabreta 7, 87–96.

Poschlod, P., Wallisdevries, M., 2002. The historical and socioeconomic perspective of calcareous grasslands - Lessons from the distant and recent past. Biological Conservation 104, 361–376. https://doi.org/10.1016/S0006-3207(01)00201-4

R Core Team. 2022. R: A language and environment for statistical computing. R Foundation for Statistical Computing, Vienna, Austria. URL https://www.R-project.org/.

Ryser, P., Langenauer, R., Gigon, A., 1995. Species Richness and Vegetation Structure in a Limestone Grassland after 15 Years Management with Six Biomass Removal Regimes. Folia Geobotanica 30, 157–167. https://doi.org/10.1007/BF02812095

Schmitt, T., 2003. Influence of forest and grassland management on the diversity and conservation of butterflies and burnet moths (Lepidoptera, Papilionoidea, Hesperiidae, Zygaenidae). Animal Biodiversity and Conservation 26, 51–67.

Shirish, P., Kelkar, S., Bhalerao, S., 2013. Mulching: A Soil and Water Conservation Practice. Research Journal of Agriculture and Forestry Sciences 1, 26-29.

Squires, V. R., Dengler, J., Hua, L., Feng, H., 2018. Grasslands of the World: Diversity, Management and Conservation. CRC Press.

Steidle, J. L. M., Kimmich, T., Csader, M., Betz, O., 2022. Negative impact of roadside mowing on arthropod fauna and its reduction with ‘arthropod-friendly’ mowing technique. Journal of Applied Entomology 146, 465–472. https://doi.org/10.1111/jen.12976

Szentes, O., 2023. Szárazság Magyarországon 2022-ben és a múltban. - Drought in Hungary in 2022 and the past. Légkör 68, 9–19.

Theenhaus A, Scheu S.,1996. The influence of slug (*Arion rufus*) mucus and cast material addition on microbial biomass, respiration, and nutrient cycling in beech leaf litter. Biol Fertil Soils 23:80–85. https://doi.org/10.1007/BF00335822

Wehner, K., Renker, C., Brückner, A., Simons, N., Weisser, W., Blüthgen, N., 2019. Land-use in Europe affects land snail assemblages directly and indirectly by modulating abiotic and biotic drivers. Ecosphere 10, e02726. https://doi.org/10.1002/ecs2.2726

Welter-Schultes, F. W., 2012. European non-marine molluscs, a guide for species identification. Planet Poster Editions, Göttingen.

Appendix

Appendix 1. Total number of living individuals in different treatment plots and periods in the two study area.

	Pretreatment			Posttreatment		
	Control	Mowed	Mulched	Control	Mowed	Mulched
Szilvásvárad	260	280	193	239	117	253
Tarnalelesz	559	903	877	447	510	595

Appendix 2. Pairwise comparison of treatments before the treatments. Posthoc tests from GLMMs separately fitted to each response variable (see the main text for details).

	Pretreatment		
Response variable	Control-Mowed	Control-Mulched	Mowed-Mulched
Number of live specimens	z = -1.39, p = 0.35	z = -0.66, p = 0.79	z = 0.75, p = 0.73
Number of species	t = -2.20, p = 0.07	t = -0.27, p = 0.96	t = 1.92, p = 0.14
Evenness	t = 0.50, p = 0.87	t = -0.56, p = 0.84	t = -1.09, p = 0.52

Appendix 3. Changes occurring in different treatment plots due to treatment. Posthoc tests from GLMMs separately fitted to each response variable (see the main text for details). Legend: Bold font indicates significant differences.

Response variable	Control	Mowed	Mulched
Number of live specimens (pre-/post-treatment)	z = 1.62, p = 0.10	**z = 4.68, p < 0.0001**	z = 1.43, p = 0.15
Number of species (pre-/post-treatment)	t = 1.53, p = 0.13	**t = 5.86, p < 0.0001**	t = 1.62, p = 0.11
Evenness (pre-/post-treatment)	t = -0.31, p = 0.76	**t = -2.39, p = 0.02**	t = 1.17, p = 0.24

Click here to access/download;Figure;Fig1_final.tiff

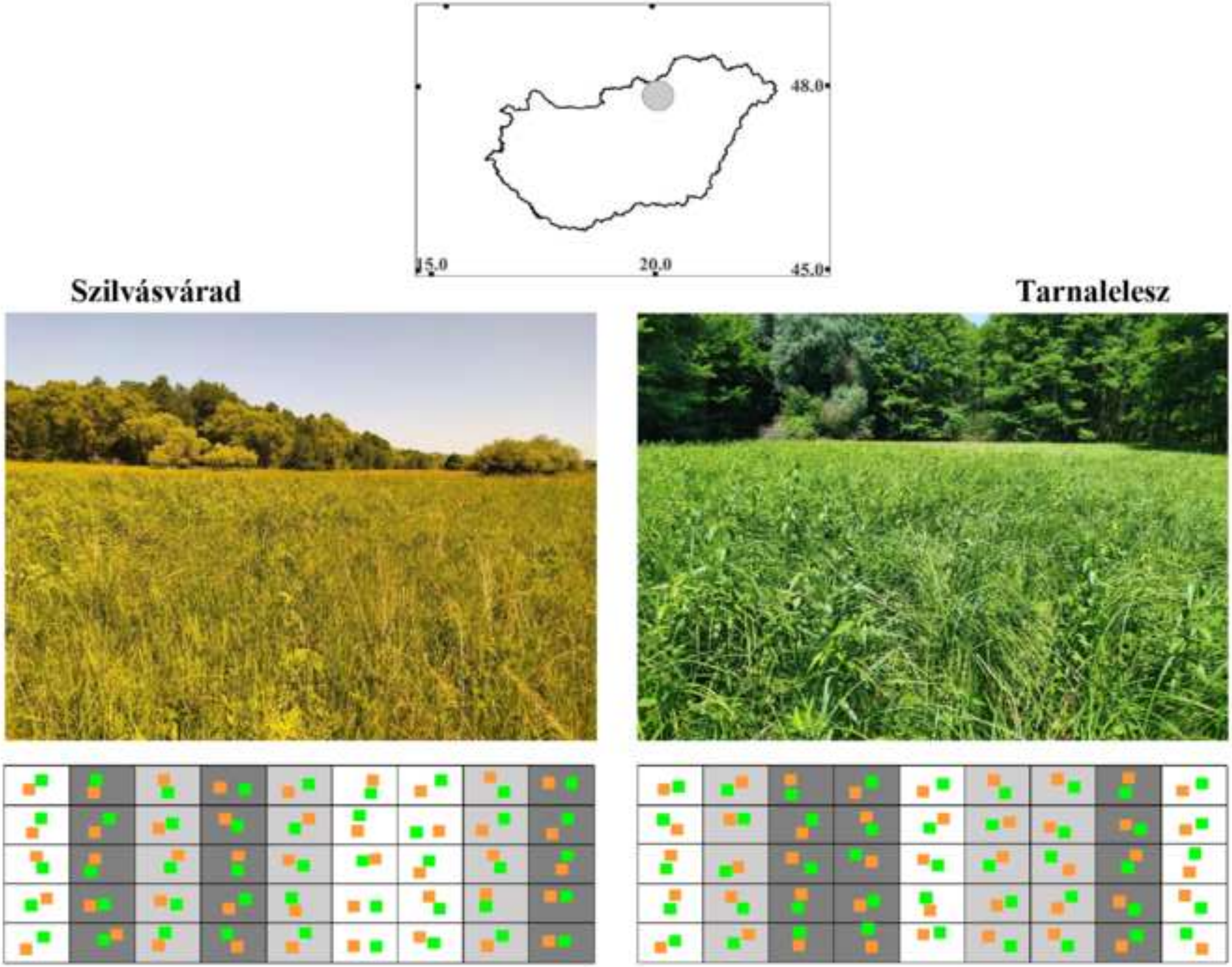

 Click here to access/download;Figure;Fig2_final.tiff

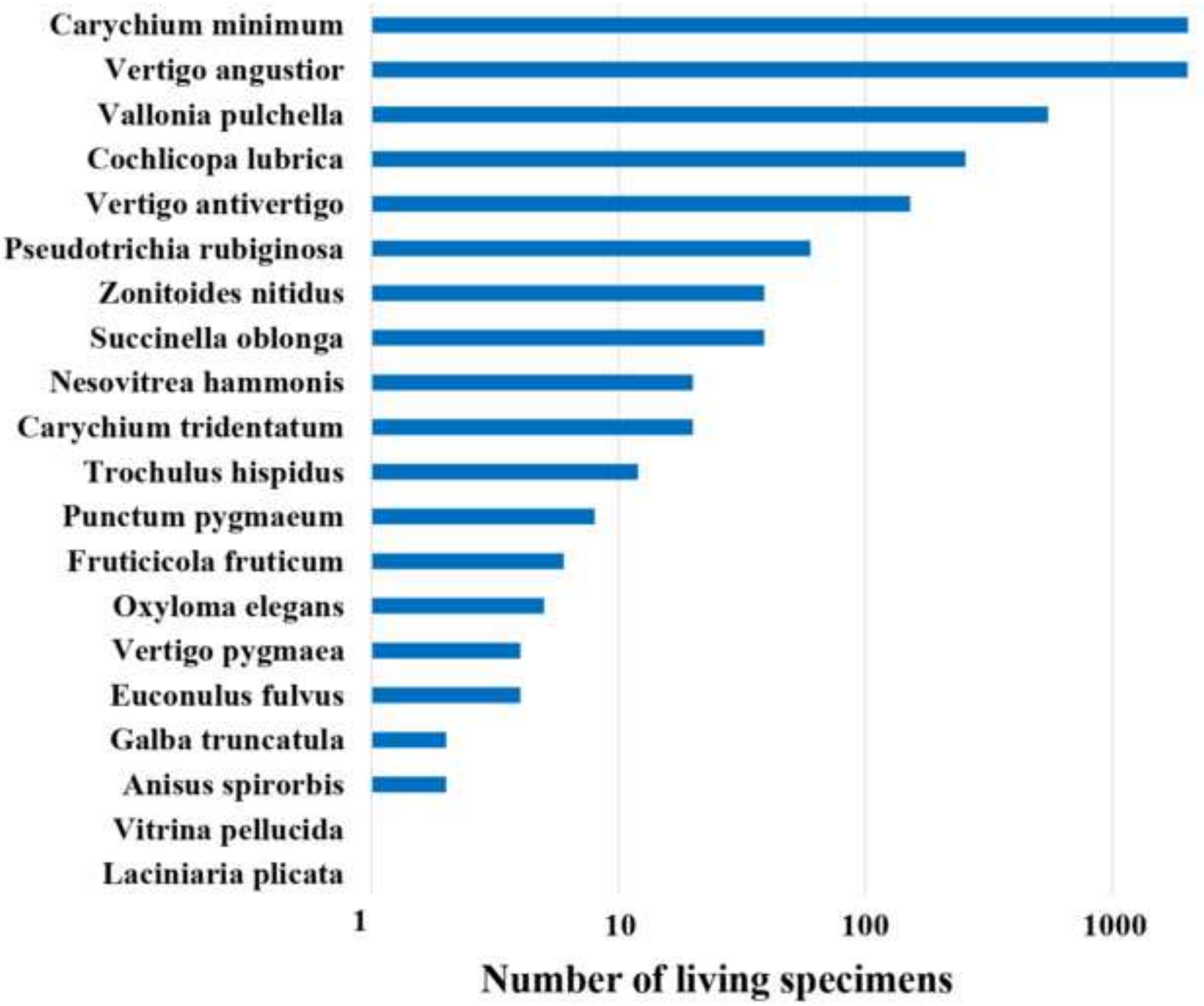

 Click here to access/download;Figure;Fig3_final.tiff

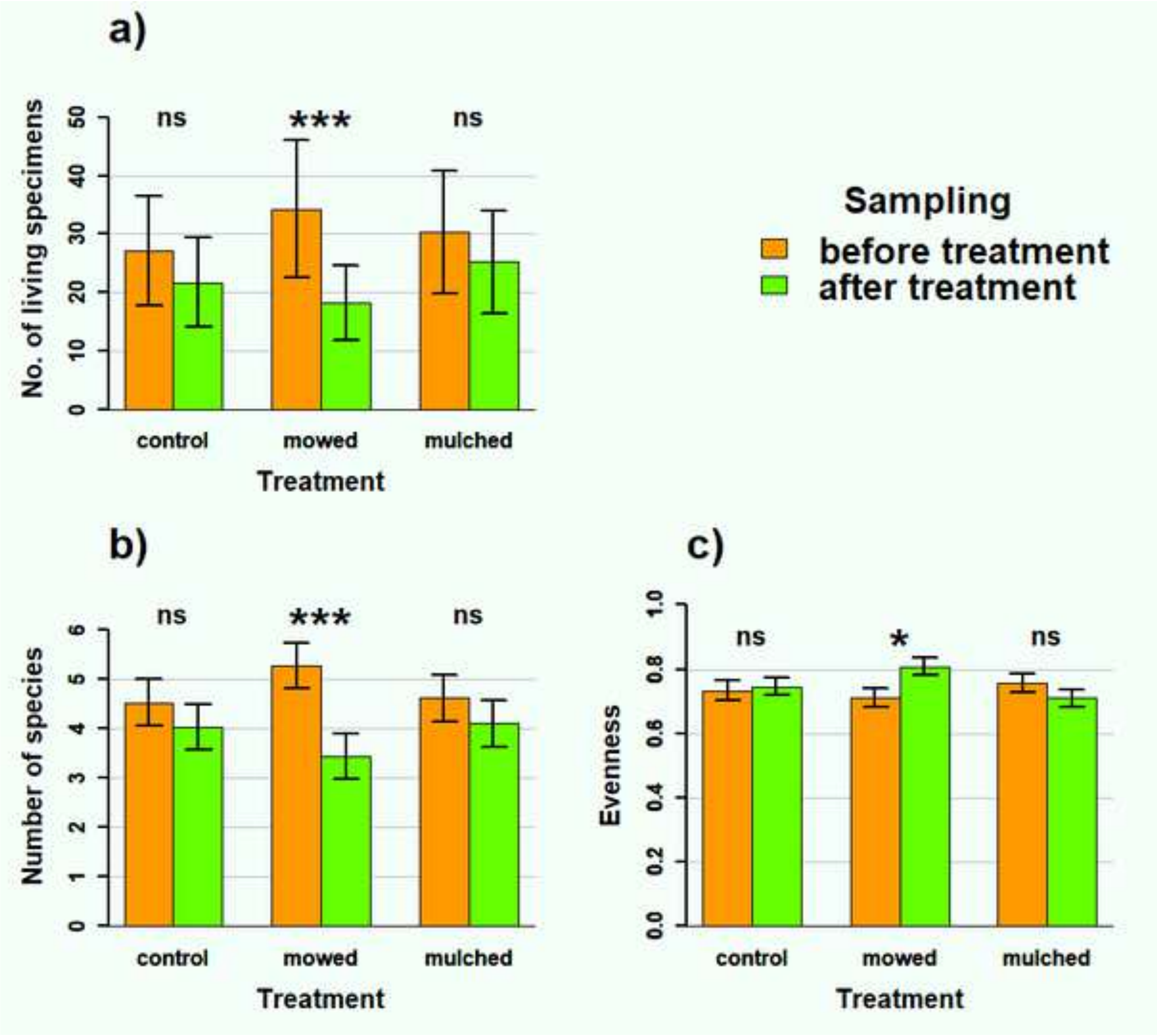

Figure

Click here to access/download;Figure;Fig4_final.tiff

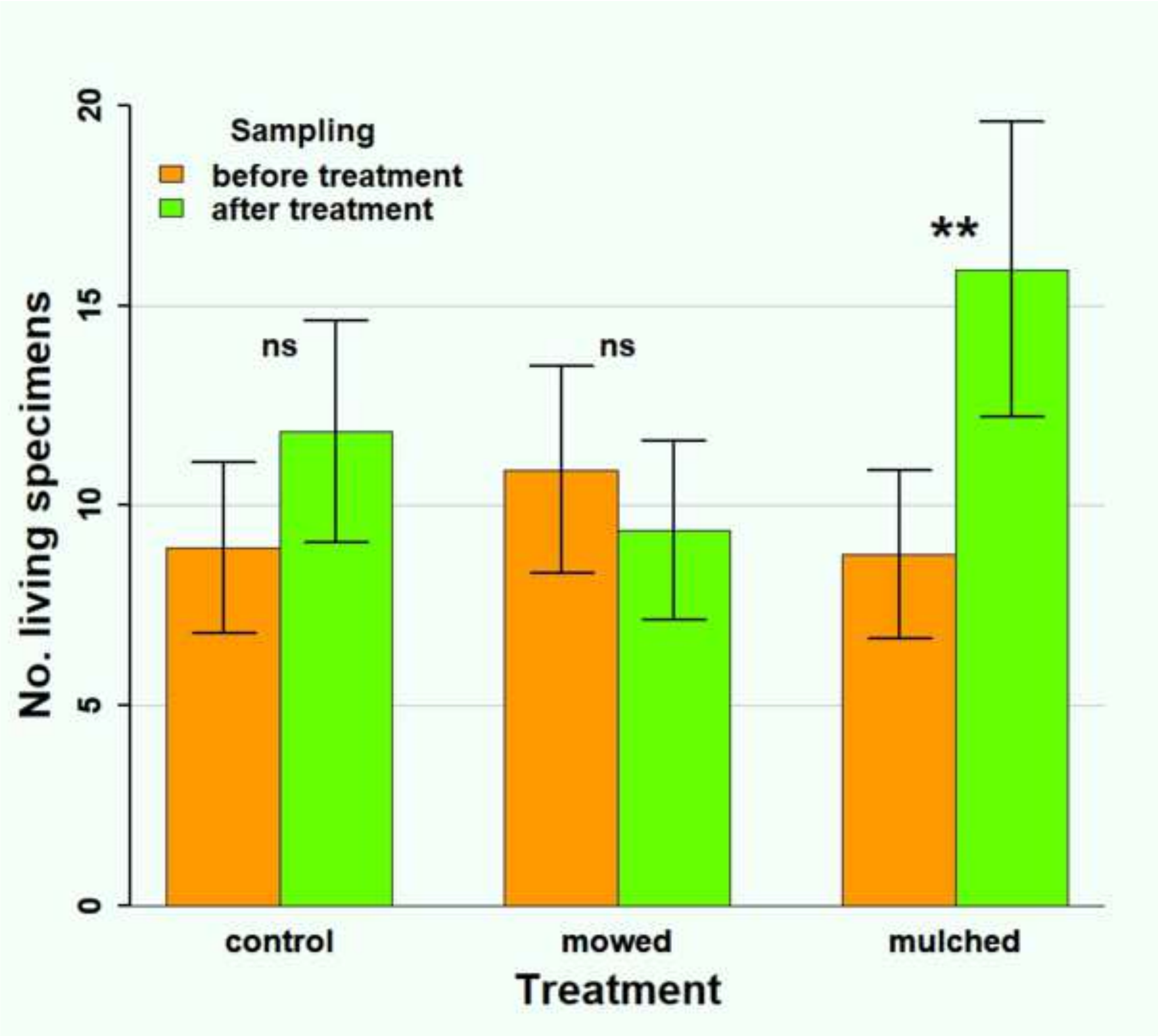

~~Experimental comparison~~The effect of mowing and mulching on snail communities: an experiment in wet meadows

Roland Farkas[1,2,3*], Miklós Bán[3], Gergő Oláh[3], György Dudás[1], Zoltán Barta[3]

[1]Bükk National Park Directorate, Eger, Hungary

[2]Juhász-Nagy Pál Doctoral School, University of Debrecen, Debrecen, Hungary

[3]HUN~~–~~-REN-DE Behavioural Ecology Research Group, Department of Evolutionary Zoology and Human Biology, University of Debrecen, Debrecen, Hungary

- Corresponding author

E-mail: ~~farkasr@bnpi.hu~~farkasro@yahoo.com

Style Definition: Normal: Don't hyphenate

Style Definition: Heading 1: Don't hyphenate

Style Definition: Heading 2: Don't hyphenate

Style Definition: Heading 3: Don't hyphenate

Style Definition: Heading 4: Don't hyphenate

Style Definition: Heading 5: Don't hyphenate

Style Definition: Heading 6: Don't hyphenate

Style Definition: Title: Don't hyphenate

Style Definition: Subtitle: Don't hyphenate

Style Definition: Header: Suppress line numbers, Don't hyphenate

Style Definition: Footer: Suppress line numbers, Don't hyphenate

Formatted

Formatted: Font: 12 pt

Formatted: Outline numbered + Level: 1 + Numbering Style: Bullet + Aligned at: 0.25" + Tab after: 0" + Indent at: 0.5"

Formatted: Font: Calibri, 11 pt

Abstract

The condition of wet meadows nowadays depends mainly on human activities; the biodiversity and productivity of these habitats can only be maintained ~~by~~through appropriate management methods. Mowing and grazing are well-known traditional methods, but a new method, regular mulching – the ~~fragmentation~~shredding of plant material and its deposition in the area - is ~~also increasingly used. The ecological impact of traditional management is still not fully known, and we know almost nothing about the effects of mulching~~becoming more widely practised. To better understand the impact of these methods we directly compared their effects on snails, an invertebrate taxon common in these habitats~~ and known as good bioindicators.~~. We experimentally manipulated~~ management methods on~~ two wet meadows in Northern Hungary, Europe, and surveyed their snail communities immediately before and ~~one year~~fourteen months after ~~treatments~~treatment. Our results showed that mowing had a detectable negative effect on the snail communities~~. On the other hand, ~~, whilst mulching did not alter their characteristics. Therefore, mulching may be a ~~more desirable method~~promising candidate for ~~short-term interventions on ~~wet ~~meadows, whilst at the same time also preserving their biodiversity~~meadow habitats.

Keywords: Gastropoda, biodiversity, abundance, management, conservation

Introduction

Grasslands~~ are an essential habitat for many animals. They make~~, making up more than 40~~.5 percent~~% of ~~land habitats~~lands worldwide~~ ~~, are home to many specialist and endemic species (Petermann and Buzhdygan, 2021). ~~Many~~Moreover, the development of ~~the species that live~~

Formatted: Font: 12 pt

Formatted: Font: 12 pt

Formatted: Font: 12 pt

Formatted: Font: 12 pt

here are specialist or endemic. In addition to their high ecological value, they are also essential economically. Indeed, human culture developed on or near was also closely linked to grasslands, which were necessaryinitially being used for, first the nomadic lifestyle of livestock grazing,animal husbandry and thenlater for the static, large-scale rearing of livestock and growing crops.forage (Squires et al. 2018). Thus, in addition to their outstanding ecological value, grasslands are also highly important economically.

The extent and condition of grasslands have changed over time; since the agricultural revolution, this has been largely in response to driven primarily by human needs and activities (Poschlod and WallisDeVries 2002). While a few activities, such as deforestation, even create new grasslands, in general their extent has decreasedgenerally shrunk at an ever-fasterincreasing rate with the spreadintensification of intensive agriculture and urbanisation. The constant need for increasing efficiency, and henceFurthermore, the emergence of new agricultural technologies, are strongly shaping has also greatly altered other characteristics of these habitats., such as landscape diversity and biodiversity (Fischer et al. 2008). TodayAs a result, the vast majority of historical grasslands have been transformed and cultivated by humans, mainly for rearing and grazing farm animals (Hejcman et al. 2013) and only small areas of pristine grassland remain. Many species of historicalhistorically endemic to grasslands were, however, able to settle in and survive on cultivated grasslands before thetheir original habitats had completely disappeared. The maintenance of today's secondary grasslands is therefore a priority task. ThusConsequently, in modern times the fate of grasslands is closely tied to humanity and their survivalgrassland communities strongly depends on their proper management by humans, which should be given a high priority.

Formatted: Font: 12 pt

Management practices are shaped by social changes, political and legal regulations, directives and agri-environmental programmes that transcend national borders, and thus are dictated by many stakeholders at various levels (Stoate et al. 2009). In recent times, fewer people are

benefiting from grasslands. The ownership of grasslands has become more centralised, with individual owners farming ever-larger areas andThe existence of wet meadows requires their agriculture is largely characterised by more intensive farming on these larger plots. (Marini et al. 2011). The adverse impact of these large-scale changes on biodiversity is shown by the negative correlation found between the species richness of a grassland and the amount of money spent on farming it (Zechmeister et al. 2003).

A specific but widespread form of grasslands are the wet meadow, defined as areas where the soil isto be saturated with water in the whole year or for at least a part of the growing season. Because they occur only underThis wetness creates conditions which significantly isolates the habitat from its surroundings, offering unique conditions for species with specific habitat requirements. Because of these specific hydrological conditions, wet meadows are highly sensitive to environmental changes. However, these hydrological conditions and are negatively affected by both current climatic changes and farming (Joyce et al. 20162016). Consequently, wet meadows are an especially vulnerable form of grasslands, which have suffered significant reduction in area across Europe over the past few centuries (Zedler and Kercher, 2005). As a result, the extent of wet meadows is decreasing in all EU countries and they are classified as threatened by the European Red List of Habitats (Jansen et al. 2016). Consequently, it is of paramount importance to investigate appropriate management methods of wet meadows.

Formatted: Font: 12 pt

The current thinking is that in general, it is desirable to maintain grasslands, rather than allowing them to return to the ancestral forested state, both because they are important for farming, but also from a conservation perspective because of their high biodiversity (Petermann and Buzhdygan, 2021). Grazing and mowing are traditional agricultural activities that are able to prevent spontaneous reforestation. Grazing is the process whereby the animals

eat the upper part of the vegetation, thus removing it from the area. In mowing, the vegetation is cut off near the surface and, after appropriate treatment, removed from the area. In the absence of these activitieshuman intervention, natural succession processes usually take place. The emergence of shrub on modern grasslands, resulting in spontaneous reforestation where the encroachment of shrubs and woody vegetation hinders farming (Moog et al. 2002) andplants threatens the continued existence of grasslands.persistence of grassland communities and can also negatively impact farming activities (Moog et al. 2002). A striking example of these processes ischanges can be seen in Central and Eastern Europe, where the political and economic changes of the late 1980's and early 1990's drastically altered the agricultural landscape. Large-scale changes in ownership and economic restructuring led to the regional abandonment of large grassland areas, many of which are still in this state today (Kuemmerle et al. 2008,; Halada et al. 2017). As grasslands harbour significant biodiversity and are important for farming (Petermann and Buzhdygan, 2021), the generally-accepted and desirable strategy is to maintain their current condition, rather than allowing them to return to an ancestral forested state.

TheTraditionally, the main methods of preventing spontaneous reforestation were grazing and mowing. More recently, the process of mechanised mulching has been developed to manage (Mašková et al. 2009) and recultivate abandoned grassland habitats (Liira et al. 2009). Despite the fact that mulching has several different meanings in EnglishUnfortunately, the specific mulching method is often not clearly defined in scientific articles (e.g. Pižl and Starý 2001,; Babálová and Štrbová 2014).), despite the fact that mulching has several different meanings in English. This methodological ambiguity makes it difficult to understand the effects of mulching on biodiversity. OneFor instance, one common meaningdefinition of mulching is the process of spreading plant material that has been harvested from another locationelsewhere, for example to protect economic crops (e.g. Bautista 2018). In other cases,

~~it~~Another is ~~used to mean~~ when plant material cut ~~in an area~~(but not shredded) by ~~mowing~~a mower machine is ~~not collected but~~ left to decompose on the spot (e.g. Liira et al. 2009, Klimeš et al. 2013). ~~In this article~~Here, we use a third ~~common~~ definition ~~of mulching~~, whereby the standing vegetation on the ~~lawn~~meadow is ~~mowed then~~ shredded ~~into small pieces~~with a flail mulcher machine, with the resulting material spread evenly over the surface of the meadow (~~and~~i.e. not removed). This definition is also used by Moog et al. (2002), Schmitt (2003), Doležal et al. (2011), Pavlů et al. (2016), Gaisler et al. (2019), and Georgi et al. (2022, 2023). Regular mulching can be used to remove ~~shrub~~shrubs and smaller trees from abandoned grasslands and restore ~~an area in a matter of~~them within a few years. It is commonly used for habitat restoration in nature conservation areas. One of the reasons for the spread of mulching is that it is more economical than traditional methods (Liira et al. 2009). Mulching requires a one-time intervention, while cutting, drying, raking, baling and transporting the bales during traditional mowing require more investment.

Formatted: Font: 12 pt

Mowing and mulching can have various effects on the environment~~.~~ and its plant and animal communities. Direct effects from the moving parts of the machines cause damage to both the flora and fauna (Humbert et al. 2010, Steidle et al. 2022). Indirect effects ~~are when~~arise from the associated changes to the physical and chemical properties of the habitat ~~are changed~~ (Humbert et al. 2009). In this case, it is important whether the plant material is removed from the area or left to decompose in place. ~~As~~When vegetation is removed, direct irradiance ~~penetrates~~can penetrate deeper, even to the surface of the soil. This can result in ~~less favourable~~higher temperatures and hence stronger evaporation compared to similar habitats with closed vegetation (Gardiner and Hassal 2009, Hesslerová et al. 2013). ~~At~~On the ~~same time~~other hand, covering the surface with cut plant material ~~results~~can result in lower temperatures (Dudas et al. 2016) and higher soil humidity (Shirish et al. 2013). Mowing and mulching also changes the soil microbiome, influencing decomposition processes and the

amount of available nutrients (Kaštovská et al. 2005, Pavlů et al. 2016, Horan et al. 2018). ~~Organic matter~~Biomass accumulated on the soil surface significantly increases the activity of decomposing organisms and enzymes in the soil (Jabran 2022).~~ It is important to understand the overall effect of these many factors in order to improve management practices.~~

Formatted: Font: 12 pt

The impact of management on grasslands depends greatly on the method used and the characteristics of the habitat concerned, and is an important research topic. Most work so far has looked at how management practices change plant communities, in particular their species composition, biomass or priority species groups. Plant communities in mowed areas have generally higher diversity than those in unmanaged, abandoned areas, except when the area is intensively ~~farmed~~managed. This has especially been demonstrated in dry, semi-arid, nutrient-poor meadows and mountain pastures (Ryser et al. 1995). In these areas, both mowing and mulching have generally been found to have ~~similar~~a positive ~~results (Zelený et al. 2001,~~ impact on maintaining species-rich habitats (Moog et al. 2002, Gaisler et al. 2004, Klimeš et al. 2013, Doležal et al. 2011). ~~However, wet meadows, a more nutrient-rich habitat, are significantly less researched. What little~~Limited evidence ~~we have indicates,~~suggests that both mowing and mulching increase species richness in wet, alluvial habitats ~~may aid habitat restoration~~ (Liira et al. 2009).

Formatted: Font: 12 pt

In contrast to our more detailed knowledge ~~of~~on the impact of mowing and mulching on flora, their effects on fauna, especially on invertebrates, ~~have received much~~has been less ~~focus~~well studied. According to the ~~current~~sparse literature, mowing mostly has a negative impact on the abundance and diversity of some invertebrate groups (snails: Pech et al. 2015, Farkas et al. 2024, less mobile spiders: Cattin et al. 2003, dung beetles: Frank et al. 2017, orthopterans: Chisté et al. 2016). Mulching has also been shown to have a similar negative effect on several groups' abundance and diversity (butterflies: Babálová and Štrbová 2014, bees and wasps:

Georgi et al. 2022, insect larvae, flower visitors: Georgi et al. 2023). However, the impact of these management methods can be influenced by a number of factors, such as the ~~time of year~~timing and the frequency of treatments (Humbert et al. 2009, Georgi et al. 2023). The literature suggests that, independently of habitat type, it is difficult to draw ~~a~~ general ~~conclusion~~conclusions about the ~~effect~~effects of grassland management on animals because different taxa living in the same habitat have been observed to respond differently to the same treatment (Pižl and Starý 2001, Cizek et al. ~~2012).~~2012). Consequently, we need more detailed studies targeted at specific groups to better understand the effects of grassland management practices.

Snails (*Gastropoda*) are common and widespread inhabitants of wet meadows (Welter-Schultes 2012). They are significant actors in the decomposition ~~process~~processes (Newell~~,~~ 1967~~). They~~): they stimulate the activity of microbial detritivores by fragmenting organic materials, and their excretion of ~~feces~~faeces and mucus production provides ~~favorable~~favourable conditions for microbial life (Theenhaus and Scheu, 1996). Furthermore, the high water content of their bodies, their slow movement and limited dispersal ability make snail populations especially sensitive to environmental change. This makes them excellent subjects for studying the effects of environmental factors in general, and impacts of management practices in particular (Pech et al. 2015, Wehner et al. 2019). Nevertheless, the impact of mulching on snail communities is ~~largely unknown. At most, conclusions can be drawn from experience with one kind of mulching when crop fields are covered with cut plant matter (Bautista et al. 2008) – but crop fields and grasslands are fairly different habitats~~unexplored.

Formatted: Font: Italic

Formatted: Font: 12 pt

The aim of our study was to identify sustainable management methods for wet meadows by using ~~snail populations~~snails as ~~indicator species. We also wanted to know whether it is possible to say after the first year that~~ a ~~treatment is harmful to the snail community. We~~

~~selected undisturbed~~target taxon group. The locations of our study were wet meadows that had remained untouched for decades. ~~We then characterized snail communities within these habitats. Using commonly utilized agricultural machinery, we~~We mowed and mulched portions of ~~the~~these areas ~~according to~~using standard agricultural machinery, following a stratified experimental design. After ~~one year~~fourteen months, we returned to collect data on the current state of the snail communities and investigated the effects of each treatment on ~~them~~their abundance and diversity.

Mowing removes biomass, while mulching leaves it in place, so we expected different effects from the two treatments. Both treatments are drastic interventions in habitats that have been abandoned for decades, so we also expected to find significant differences compared to the control areas. We chose the fourteen-months interval to investigate the effects of treatments because early recognition of adverse impacts is important to prevent valuable habitats from being treated with harmful methods in the longer term.

Methods

Formatted: Font: 12 pt

Study area

Formatted: Font: 12 pt

The experiment was carried out at two sites in Northern Hungary, Europe (**Fig 1**). ~~Both of them~~The central WGS84 coordinates of the sites are the following: Szilvásvárad X= 48.1250, Y= 20.3920; Tarnalelesz X= 48.0908, Y= 20.1506. Both sites are located in stream valleys of a hilly area, west of the Bükk Mountains. This area is characterised by high forest cover, with wet meadows occurring almost exclusively along the valley bottom watercourses. Annual rainfall in this area ranges between 600-700 mm and the average annual temperature is around 9°C (Dövényi 2010). In the valley bottoms meadow moulding soils have developed on clayey, loamy, sandstone bedrock (Dövényi 2010). In both sites, the habitats studied were non-

tussock tall-sedge beds, with a dominance of sedge (*Carex acutiformis*). The characteristic associated species observed were *Lysimachia vulgaris*, *Lythrum salicaria*, *Cirsium oleraceum* and *Urtica dioica*. Both sites were flat, horizontal areas ~~without any obvious microhabitat~~with an apparently uniform structure. No bushes or trees were present in any of the sites. An important criterion for the selection of the sites was that they represent wet meadows that have not been farmed for the last ~~20~~ 30 years. To ~~determine~~verify this, a series of archive aerial photographs (www.fentrol.hu) were used.

Formatted: Font: 12 pt

Figure 1. The ~~map of the~~ study sites.

Legend: Top ~~center: the borders~~centre: a map of Hungary ~~are visible, and~~with a ~~gray~~grey circle ~~indicates~~indicating the location of the study areas. ~~In the bottom row, detailed maps depicting the locations of sample sites (orange dots) are presented. Left side: Tarnalelesz sample site, right side: Szilvásvárad sample site. Both detailed maps share the same scale and orientation.~~ WGS 84 geographic coordinates are marked on the axes. ~~Map data: © OpenStreetMap contributors, SRTM | Map display: © OpenTopoMap (CC-BY-SA).~~Middle: photos of the habitats before the management. Bottom: detailed sampling design. Columns represent treatment plots, different colours indicate the type of treatment: white – control, light grey – mowed, dark grey – mulched. Randomly placed squares symbolize sampling points: orange - pretreatment, green – posttreatment.

Experimental design

Formatted: Font: 12 pt

A sample area of 45x15 ~~metres~~meters was marked out in each site. These were divided into 9 ~~parallel parcels~~plots of 5 x 15 ~~metres~~meters each. Both the edges of the sample area and the boundaries of the ~~parcels~~plots were marked with permanent stakes. Out of the 9 ~~parcels~~plots,

3 were mowed, 3 were mulched, and 3 remained untouched as ~~control plots.~~controls. Treatments were assigned to the ~~parcels in a stratified random way: each block of three consecutive parcels received all three treatments~~plots in a random order. ~~In every parcel, one sampling point~~Each plot was ~~assigned to~~ divided into 5 sections of 5x3 meters. In each ~~5x3 meter plot~~of these, a sample was taken from a randomly selected point, resulting in 45 sampling points at each site (~~**Fig 2**~~)

15 per

~~Figure 2. Detailed sampling design.~~

~~Legend: a) Szilvásvárad site, b) Tarnalelesz site. The columns represent~~ treatment ~~parcels, and the rows indicate sampling plots. Different colors indicate the type of~~) (**Fig 1**). This gives n=30 replicates per treatment~~: white – control, light gray – mowed, dark gray – mulched.~~ for the whole study (2 study sites x 3 treatment plots per site x 5 samples per plot).

On June 28, 2021. (Szilvásvárad) and June 29, 2021. (Tarnalelesz), a malacological sampling ~~was taken in~~took place at each ~~plot~~sampling point before the start of management. Treatments were carried out ~~once~~ a few days after this sampling, on July 1, 2021 at both sites. This is a typical time for farmers in the region to carry out farming activities on meadows. In the mowed ~~parcels~~plots, we cut the vegetation with a disc mower machine pulled by a tractor. The cut material was immediately removed from the surface using hand rakes. Mulching treatment was done by a ~~mulching~~flail mulcher machine pulled by a tractor to shred the vegetation into pieces smaller than 10 cm. The resulting material was not removed and evenly covered the surface. Both sites were then left undisturbed ~~for approximately one year.~~. Subsequently, on August 15, 2022 (Szilvásvárad) and August 14, 2022 (Tarnalelesz), we went back to the sites and again collected a malacological sample from each ~~plot.~~sampling point. Malacological sampling was carried out in the morning hours each time. Sampling dates were

Formatted: rynqvb, Font: Calibri, 11 pt, English (United States)

Formatted: rynqvb, Font: Calibri, 11 pt, English (United States)

Formatted: rynqvb, Font: Calibri, 11 pt, English (United States)

Formatted: Space Before: 0 pt, After: 0 pt

Formatted: Font: 12 pt

selected to ensure that the weather conditions were similar and rain-free during the two-day periods.

One malacological sample was taken per ~~plot per~~ sampling point on each occasion. Sampling was performed by collecting the leaf litter and the top 1 cm thick layer of the soil from a 25X25 cm quadrat. The collected samples were stored in airtight plastic bags in a cool, dark place. On the second day after collection, samples were floated~~. They~~: they were ~~kept~~poured individually ~~in~~into buckets containing a volume of water significantly greater than the sample volume and left for half an hour. ~~Immediately thereafter, the~~ The floating material~~, mainly of vegetable origin,~~ was then washed thoroughly in the buckets to remove any ~~remaining~~trapped snail shells~~. The washed, snail-free floats were~~ and discarded. The material remaining in the ~~dish~~bucket, including the snails, was washed through a sieve with a mesh size of 0.75 mm. The hole size was chosen so as not to allow the passage of the smallest adult snail specimens. The material remaining on the sieve contained the washed snail shells and other small organic and inorganic debris. This was immediately placed in 70% ethanol and stored.

Later, the snail shells were removed from these samples using a 3.5X magnifying glass. The sorted snail shells were identified to the species level using a stereo microscope (Motic SMZ-168 series, 10X magnification). All determinations were carried out by RF, and they were based on the works of ~~Welter-Schultes (2012),~~ Kerney et al. (1983~~) and~~), Glöer and Meier-Brook (2003~~).~~) and Welter-Schultes (2012). Only fully developed and intact (not broken or incomplete) shells of live adults were ~~used~~counted in the study. Live specimens were considered to be those in which the body of the snail could be observed inside the translucent shell or by looking into the aperture of the specimens. Slugs were not included in the study because this sampling method is not suitable for their quantitative analysis (Cameron and Pokryszko, 2005).

Formatted: Font: 12 pt

Statistical analysis

Formatted: Font: 12 pt

All statistical analyses were performed in the R ~~interactive~~ statistical environment (v4.2.2, R Core Team 2022).

Formatted: Font: 12 pt

~~Snail communities were characterised~~First, we took a community based approach by ~~(i)~~calculating the ~~total number of specimens, (ii)~~Bray-Curtis distance matrix between the ~~number of species and (iii) the normalised Shannon–Weaver diversity: where the diversity measure normalised~~sampling points by the ~~maximum possible diversity, ln R (Shannon~~vegdist function of the vegan package (ver. 2.6-8, Oksanen et al., 2024) and then partitioned the variance of the distance matrix by performing a PERMANOVA (McArdle and ~~Weaver, 1949~~Anderson, 2001) with sampling time (pre- and posttreatment), treatment (control, mowing and mulching) and their interaction as explanatory terms (adonis2 function of vegan). If the different treatments differently affect the changes in snail communities over time one would expect a significant interaction between the variables of treatment ~~(control, mowing and mulching) and sampling time (pre- and post-treatment). To statistically investigate this possibility, we~~and sampling time. PERMANOVA performs random permutations of the data to derive the distributions of estimated parameters. In our model, spatial non-independence (i.e. data come from two separate areas) was controlled for by restricting permutations to within areas only (Bakker 2024).

Snail communities were further characterised by three community characteristics: (i) the total number of specimens, (ii) the number of species and (iii) Pielou's species evenness, H' = $\Sigma p_i \ln p_i / \ln S$, where p_i is the relative abundance of species i in the sample and S is the number of species in the sample (Pielou 1975). We fitted three generalised linear mixed effects models (GLMMs) to our data, one for each community characteristic. We entered treatment,

sampling time and their ~~two-way~~ interaction as fixed effects. We also took into account the statistical non-independence caused by spatial proximity by entering random effects into the models. Random effects were entered as sampling point ID nested within ~~parcel~~plot ID nested within ~~area~~site ID. Note~~, however~~, that we had to remove the random effect terms from the model for ~~Shannon diversity~~evenness, because this model did not converge as the variance explained by the random effect terms was essentially zero. We used a negative binomial error distribution for the number of specimens, and normal error distributions for the number of species and ~~diversity~~evenness. Inspecting the model fits with the DHARMa R package (v0.4.6, Hartig 2022) did not indicate any deviations from model assumptions.

We also analysed how treatments affected the number of individuals for the three most common species in the samples (*Carychium minimum*, *Vertigo angustior* and *Vallonia pulchella*). We fitted separate GLMM models for each species data with the same fixed and random effects as specified above. We used a negative binomial error distribution for all three species. Model assumptions were also checked by the DHARMa package.

~~Models~~All GLMM models were fitted with the 'glmmTMB' function of the glmmTMB package (v1.1.7, Brooks et al. 2017). If a significant interaction was detected (by log ratio tests) we tested posthoc which treatment groups were significantly different from each other by the emmeans function of the emmeans package (v1.8.4-1, Lenth 2023).

Results

Formatted: Font: 12 pt

During the study, 5233 living individuals of 20 species were found in the two sample sites combined~~ **(Table 1)**.~~. We show the total number of individuals by species is shown in **Fig2**.

Total number of living individuals in different study sites, treatment parcelsplots and periods are givensampling times is shown in **Appendix 1**.

Formatted: Font: Not Bold

Formatted: Space Before: 0 pt, After: 0 pt

Figure 2**Species**	**Number of live individuals**
Anisus spirorbis	2
Carychium minimum	2031
Carychium tridentatum	20
Cochlicopa lubrica	255
Euconulus fulvus	4
Fruticicola fruticum	6
Galba truncatula	2
Laciniaria plicata	1
Nesovitrea hammonis	20
Oxyloma elegans	5
Pseudotrichia rubiginosa	60
Punctum pygmaeum	8
Succinella oblonga	39
Trochulus hispidus	12
Vallonia pulchella	551
Vertigo angustior	2021
Vertigo antivertigo	152
Vertigo pygmaea	4

Vitrina pellucida	1
Zonitoides nitidus	39

Table 1. Total number of living individuals in the samples.

Formatted: Font: 12 pt

Before Numbers of individuals are plotted on a logarithmic scale. Species are shown with decreasing numbers of individuals from top to bottom.

PERMANOVA analysis indicated a significant interaction between sampling time and treatment, we found ($F_{2,169}$ = 2.209, p = 0.003, R^2 = 0.023) suggesting that communities changed differently with different treatments. Investigating the fitted parameters of the PERMANOVA model revealed (i) no initial differences between the communities of different treatments ($F_{2,169}$ = 1.033, p = 0.251, R^2 = 0.010), (ii) significant changes with sampling time ($F_{1,169}$ = 17.619, p = 0.001, R^2 = 0.091) and that (iii) only the mowed community changed differently from the control ($F_{1,169}$ = 2.921, p = 0.006, R^2 = 0.015).

Analysing community characteristics (abundance, species richness and evenness) with GLMMs, there were no significant differences between the mowed, mulched and control parcelsplots before treatment in either the number of live individuals, number of species or Shannon diversity valuesany characteristic (**Appendix 2**).

Looking next at the effects of treatmentHowever, we found a significant interaction between treatment and sampling time for all community characteristics: number of living individuals (χ_2^2 = 6.99, p = 0.03), species number (χ_2^2 = 11.32, p = 0.01) and Shannon diversityevenness

(χ_2^2 = 6.26, p = 0.04). This indicates that different treatments affected the snail communities differently.

Next, by comparing treatment groups, we examined how snail populations changed due to ~~each treatment (~~the treatments (**Fig 3, Appendix 3**~~**, Fig 3**~~). The estimated number of living individuals decreased in all three management types, but this decrease was only significant in the mowed areas, indicating that the mowing itself likely caused a ~~strong~~ decrease. In terms of species numbers, ~~the treatment also~~again only mowing caused a significant decrease~~ in the mowed areas.~~. For ~~diversity~~evenness, we also found a significant difference only in the mowed areas, but in this case ~~diversity~~the evenness increased~~ as a result~~, meaning that the abundance of ~~mowing~~each species in the community became more similar.

Figure 3. The impact of treatment on three community characteristics

The impact of treatment a) on the number of living individuals, b) on the number of species and c) on ~~diversity~~evenness. Vertical error bars indicate standard errors of the estimates derived by the emmeans function from the fitted GLMM models (***: p<0.001, *: p<0.05, ns: p>0.05).

~~In~~Finally, in addition to ~~community-level effects~~examining entire communities, we investigated whether treatments ~~had species-level effects on~~affected the abundance of the three most common species (*Carychium minimum*, *Vallonia pulchella*, *Vertigo angustior*). In *Carychium minimum* the abundance decreased significantly with sampling time (χ^2_1 = 97.16, p < 0.001) independently of treatment (effect of treatment: χ^2_2 = 0.72, p = 0.696; interaction:

Formatted: Font: 12 pt

Formatted: Superscript

Formatted: Subscript

Formatted: Superscript

Formatted: Subscript

χ^2_2 = 2.85, p = 0.241). In the case of *Vallonia pulchella* ~~none of the effect of~~ neither treatments (χ^2_2 = 3.55, p = 0.169~~) and~~), sampling times (χ^2_1 = 2.62, p = 0.105~~) neither of~~), nor their interaction (χ^2_2 = 3.98, p = 0.137) ~~was significant.~~significantly affected abundance. Only ~~in the case of~~for *Vertigo angustior*~~, we found that~~ did treatments (interaction: χ^2_2 = 7.90, p = 0.019) differently affect the ~~change in number of individuals~~abundance; mulching ~~(posthoc test: z = -3.25, p = 0.001)~~ significantly increased the number of individuals (posthoc test: z = -3.25, p = 0.001) while the other two treatments did not have such ~~an~~any effect (control, posthoc test: z = -1.53, p = 0.127; mowing, posthoc test: z = -0.81, p = 0.417; **Fig 4**).

Formatted: Superscript

Formatted: Subscript

Figure 4. The impact of treatment on the number of living *Vertigo angustior* individuals. Vertical error bars indicate standard errors of the estimates (**: p<0.01, *: p<0.05, ns: p>0.05).

Formatted: Font: 12 pt

Discussion

Formatted: Font: 12 pt

We found that ~~different treatments had different impacts on the~~ snail communities~~. In the~~ declined significantly in mowed areas, ~~negative changes in the snail community were observed one year after the treatments. Significant declines in both live individuals and species richness indicate that mowing had a~~ but not mulched ones, indicating that mowing can have a detectable negative impact on snail communities~~ even after one year. In comparison, mulched and control areas experienced no significant changes.~~

~~Our~~ . This seemingly contradicts our finding that ~~diversity~~ Pielou’s evenness increased on mowed ~~plots might seem to contradict our claim that mowing can have a negative effect on snail communities. Under~~areas. However, the two are not necessarily mutually exclusive,

since under certain conditions, ~~however,~~ diversity ~~index~~indices can increase despite a decrease in number of individuals and species. For instance, if ~~more~~ the decrease is larger in more abundant species ~~then~~the distribution of individuals among species becomes more ~~even and hence the diversity index increses~~uniform and evenness increases. In cases like this, we must define our optimal outcome, and it seems sensible to choose a smaller decrease in abundance as more optimal than an increase in evenness. So in this situation, our finding remains, that mowing has negative effects on snail communities whereas mulching does not.

There has been relatively little research on the effects of mowing on snails, and, as far as we know, there is no previous work ~~on~~regarding the effects of mulching ~~on them~~. Previous ~~malacological~~ research ~~on mowing~~ has mainly focused on longer-term effects over several years ~~long effects~~. For instance, Pech and colleagues (2015; working in oligotrophic grasslands), and Farkas and colleagues (2024; on valley-bottom wet meadows) found long-term reductions in abundance and species numbers due to mowing, but Wehner and colleagues (2019; over a wide range of meadows) found an increase in snail community diversity in mowed areas, while no difference in abundance was found. All three studies are similar in that they were conducted on areas that were previously mowed (albeit for varying durations), while our current study was conducted in wet meadows that were previously untreated for decades. In addition, the above studies investigated the effects of mowing not by comparing samples taken before and after treatment on the same site, but by analyzing data from mowed and adjacent unmowed areas. Despite these methodological differences, our result on the detrimental effects of mowing is similar to two of these previous studies.

Our findings pertain to snail communities, yet wet meadows harbor numerous other abundant invertebrate taxa, which may respond differently to different management practices. Mowing had negative effects on abundance of less mobile spiders (Cattin et al. 2003),

Formatted: Font: Bold

Formatted: Border: Top: (No border), Bottom: (No border), Left: (No border), Right: (No border), Between : (No border)

Formatted: Font: 12 pt

Formatted: Font: 12 pt

~~orthopterans~~species richness (Cizek et al. 2012~~,~~) and diversity (Chisté et al. 2016) of orthopterans, and biomass of dung beetles (Frank et al. 2017). Other studies have found ~~negative effects of~~ that mulching ~~on moth~~decreased abundance of herbivorous insect larvae, flower visitors (Georgi et al. 2023) and herbivore-hunting wasp (Georgi et al. 2022) communities. ~~There are studies that conclude that mulching is less beneficial than mowing for earthworms, oribatids (~~Pizl and Stary (2001~~), and~~) conclude that abundance of earthworms is lower on mowed than mulched sites, Schmitt (2003) found that the abundance of butterflies ~~(Schmitt 2003).~~is higher in mowed than mulched plots. However, the number of studied invertebrate groups is limited. This makes it difficult to ~~generalize~~draw general conclusions about the effects of treatments on entire invertebrate communities ~~of~~in a particular habitat. Moreover, the range of habitats studied is broad, with varying initial conditions: some studies monitor the effects of different ongoing treatments (Schmitt 2003, Chisté et al. 2016, Frank et al. 2017~~, Schmitt 2003~~), while others established new experimental setups on habitats that ~~have~~had earlier undergone some form of regular treatment (Georgi et al. 2022, 2023). However, there are no previous studies where the initial state of the habitat, like ours, was untreated for decades. ~~Going forward, significantly more research is needed to investigate the effects of grassland management on invertebrates in order to ensure that different habitats receive the most suitable management practices for the widest possible range of taxa.~~

Our results indicate that members of snail communities did not respond uniformly to the management. Of the three most common species we examined, ~~only *Vertigo angustior* showed a treatment specific response. Both~~both *Carychium minimum* and *Vertigo angustior* are ~~typical of~~limited to wetland habitats, but only the latter was affected by the management. *Vallonia pulchella* has a wider range of habitat preferences, and was also unaffected by the treatment. Similar results were observed in management studies with other taxa. Cizek and

Formatted: Font: 12 pt

Formatted: Font: 12 pt

Formatted: Font: 12 pt

colleagues (2012) found that the abundance of different ground beetle species changed differently with the same treatment. Pižl and Starý (2001) showed differential changes in abundance for different species of oribatids living above the soil surface in response to management. Both sources agree that generalist species tolerate changes better than specialists. *Vertigo angustior* is still considered a common species in Hungary, but its distribution in Western Europe has drastically declined, which can be attributed to changing and increasingly intensive agriculture (European Environment Agency 2013). Therefore, the appropriate choice of management practices is of paramount importance ~~in~~for its ~~case~~preservation.

Formatted: Font: 12 pt

~~Our study was designed to examine the one year effects of a single intervention. Negative effects of mowing were already detectable one year after treatment. We assumed that no significant changes in the chemical and soil properties of the site could have occurred in such a short time. The one year negative effects on snail communities at both sites for all treatments might have been mainly due to effects such as sudden increase in irradiance, increases in temperature, decreases in humidity and organic matter.~~

~~Management~~The observed consistent declines in abundance of snail communities at both sites for all treatments were likely due to historic drought and heatwaves occurred in Hungary in 2022 (Szentes 2023). In addition to these changes, management methods can have both direct (e.g. caused by the machines themselves) and indirect effects (e.g. caused by habitat transformation) on invertebrate communities. In our case, the changes observed in the mowed snail community are most probably due to indirect effects. The majority of the snail species studied live in the litter layer of the habitat, and the rotating parts of the equipment do not affect this layer. Trampling by tractor wheels also has a direct negative effect on species living on the ground (Humbert et al. 2010) but in our study the mowed and mulched plots were trampled to the same extent. Instead, the significantly higher decrease in abundance

from mowing might have been mainly due to effects such as the sudden increase in irradiance and temperature, and a decrease in humidity (Shirish et al. 2013, Dudas et al. 2016).

In our study, mowing was defined as cutting the vegetation and immediately removing the biomass, which is different to real-life agricultural, practices usually have an effect due to changes in habitat characteristics (Wehner et al. 2019). Over a longer period of time, habitat characteristics to which snail or other invertebrate communities were originally adapted can change significantly. Sensitive species may recede. In practice, having cut it, farmers typically leave the biomass remains on the surface for days, while others, adapting to new opportunities, may advance. it is dried, turned, windrowed, and finally baled and removed. Leaving the cut hay temporarily out might prevent desiccation, thus reducing the negative effects of mowing. However, in nature conservation treatments focused on small areas, the immediate removal of the cut biomass is standard practice. Therefore, our study better represents current nature conservation, rather than agricultural, practices.

Formatted: rynqvb, Font: Calibri, 11 pt, English (United States)

Formatted: rynqvb, Font: Calibri, 11 pt, English (United States)

Careful selection of management conditions can significantly reduce their negative impact on invertebrates.flora and fauna. These include staggering managementstreatments throughout the year or leaving parts of the habitat unmanaged (Pech et al. 2005, Cizek et al. 2012,).). The negative effects of treatments could also be prevented or reduced by tailoring them to the life cycle of invertebrates (Georgi et al. 2022, 2023). It may also be beneficial not to manage an area every year, but this can threaten the survival of the whole habitat afterbeyond a certain reduction in intensity (Ryser et al. 1995, Moog et al. 2002).

Our experimental study had promising results, but we are still far from having sufficient knowledge of the effects of grassland treatments. Our research was limited to one season, two sites and a comparison of three treatment methods. Short-term results are important because

they can guide future treatments. On the other hand, to achieve sustainability, longer-term studies are needed. Our current knowledge in this area is still limited, and it would be useful to expand in multiple ways. First, by including additional litter or soil-dwelling taxa, in order to get a more complete idea of the effects of each treatment on entire ecosystems. Second, by comparing both mowing and mulching as well as their common combinations, since in practice, management methods are often applied together, resulting in potentially different effects than just one treatment alone. Third, including different spatiotemporal patterns and intensities of treatments would allow us to even better understand the effects of real-world agricultural practices.

To summarise, significantly more research is needed to properly understand the effects of grassland management on flora and fauna. This would allow us to optimise real-world agricultural practices to each habitat, ensuring the most suitable management practices are used and thus best preserving biodiversity.

Conclusions

Wet meadows have been ~~sites of grassland management~~managed and cultivated by humans since ancient times. ~~The evolution of~~New methods and increasingly intensive agricultural practices are significantly transforming these valuable habitats. While the impact of treatments on vegetation is somewhat understood, little is known about the fate of animal communities, especially ~~invertebrates.~~invertebrate ones. Through a controlled grassland management experiment, we examined the effects of mowing and mulching on the snail communities of wet meadows. Our results indicate that significant changes ~~are~~can be observed ~~just one year~~fourteen months after ~~initiating~~ treatment. The negative impact of mowing was ~~clearly~~ evident within this time frame, while mulching did not lead to significant changes. Mulching thus seems to be a ~~practical~~suitable way for short-term interventions of

grassland areas without the negative effects of mowing~~.~~ on snail communities. Sustaining grasslands in the long term requires ~~attention~~ not only ~~to~~ maintaining their habitat ~~maintenance~~structure but also ~~to~~ preserving their quality and biodiversity. ~~Therefore~~Given the observed negative effects of mowing, selecting appropriate methods is crucial. Within a given area, numerous taxa are present, each responding differently to interventions. Hence, conducting similar studies across ~~many and~~ diverse ~~invertebrate~~animal groups is necessary. In addition, whilst ~~one year conclusions regarding~~data on the effects of different management methods after one year are useful, there is still a lack of long-term monitoring of grassland management, particularly regarding mulching~~.~~ on wet meadows. Initiating, supporting~~,~~ and maintaining more ~~such~~ long-term research projects is crucial. In addition to economic considerations, ecological aspects of meadow management must also be taken into account to ensure sustainability for the future.

~~Acknowledgement~~

~~We are grateful to Tamás Székely, Jr. for his help to correct our English.~~

Acknowledgement

We are grateful to Tamás Székely, Jr. for his comments on the manuscript. The study was prepared with the professional support of the Doctoral Student Scholarship Program of the Co-operative Doctoral Program of the Ministry of Innovation and Technology financed from the National Research, Development and Innovation Fund (Hungary). The study was supported by a National Research, Development and Innovation Office grant (NKFIH K138503 to ZB). ZB was supported by a National Research, Development and Innovation Fund grant (TKP2021-NKTA-32).

Literature

Babálová, M., Štrbová, E., 2014. Influence of grasslands management on diversity of the diurnal butterflies of Vlkolínec Village (Central Slovakia). Ecology and noospherology 83–91.

Bakker, Jonathan D. 2024. Applied Multivariate Statistics in R. University of Washington. https://uw.pressbooks.pub/appliedmultivariatestatistics/.

Bautista, F., Delgado, M., Estrada-Medina, H., 2008. Effect of legume mulches and cover crops on earthworms and snails. Tropical and subtropical agroecosystems 8, 45–60.

Brooks, M. E., Kristensen, K., van Benthem, ~~KJ~~K. J., Magnusson, A., Berg, ~~CW~~ C. W., Nielsen, A., Skaug, ~~HJ~~H. J., Maechler M., Bolker, ~~BM~~B. M. 2017. glmmTMB Balances Speed and Flexibility Among Packages for Zero-inflated Generalized Linear Mixed Modeling. The R Journal, 9(2), 378-400. doi: 10.32614/RJ-2017-066.

Cattin~~, M.-F.,~~ Blandenier, ~~G~~M.-F., Banašek-Richter, C., Bersier, L.-F., 2003. The impact of mowing as a management strategy for wet meadows on spider (Araneae) communities. Biological Conservation 113, 179–188. https://doi.org/10.1016/S0006-3207(02)00297-5

Chisté, M. N., Mody, K., Gossner, M. M., Simons, N. K., Köhler, G., Weisser, W. W., Blüthgen, N., 2016. Losers, winners, and opportunists: How grassland land-use intensity affects orthopteran communities. Ecosphere 7, e01545. https://doi.org/10.1002/ecs2.1545

Cizek, O., Zámečník, J., Tropek, R., Kocarek, P., Konvicka, M., 2012. Diversification of mowing regime increases arthropods diversity in species-poor cultural hay meadows. J Insect Conserv 16, 215–226. https://doi.org/10.1007/s10841-011-9407-6

Doležal, J., Mašková, Z., Lepš, J., Steinbachová, D., de Bello, F., Klimešová, J., Tackenberg, O., Zemek, F., Květ, J., 2011. Positive long-term effect of mulching on species and functional trait diversity in a nutrient-poor mountain meadow in Central Europe. Agriculture, Ecosystems & Environment, Understanding species and community response to

Formatted: Font: 12 pt

Formatted: Font: 12 pt

Formatted: Font: 12 pt

Formatted: Font: 12 pt

Formatted: Font: 12 pt

Formatted: Font: 12 pt

environmental change - a functional trait perspective 145, 10–28. https://doi.org/10.1016/j.agee.2011.01.010

Formatted: Font: 12 pt

Dövényi, Z. (ed.), 2010. Magyarország kistájainak katasztere. MTA Földrajzi Kutatóintézet, Budapest.

~~Dudas~~Dudás, P., ~~Menyhart~~Menyhárt, L., Gedeon, C., Ambrus, G., ~~Toth~~Tóth, F., 2016. The effect of hay mulching on soil temperature and the abundance and diversity of soil-dwelling arthropods in potato fields. Eur. J. Entomol. 113, 456–461. https://doi.org/10.14411/eje.2016.059

Formatted: Font: 12 pt

European Environmental Agency, 2013. Vertigo angustior -– Report under the Article 17 of the Habitats Directive Period 2007-2012. https://projects.eionet.europa.eu/habitat-art17report/library/2007-2012-reporting/factsheets/species/molluscs/vertigo-angustior/download/en/1/vertigo-angustior.pdf

Formatted: Font: 12 pt

Farkas, R., Bán, M., Barta, Z., 2024. Mowing wet meadows reduces the health of their snail communities. PeerJ 12:e16783 DOI 10.7717/peerj.16783

Formatted: Font: 12 pt

Frank, K., Hülsmann, M., Assmann, T., Schmitt, T., Blüthgen, N., 2017. Land use affects dung beetle communities and their ecosystem service in forests and grasslands. Agriculture, Ecosystems & Environment 243, 114–122. https://doi.org/10.1016/j.agee.2017.04.010

Formatted: Font: 12 pt

Gaisler, J., Hejcman, M., Pavlů, V., 2004. Effect of different mulching and cutting regimes on the vegetation of upland meadow. Plant Soil Environ. 50, 324–331. https://doi.org/10.17221/4039-PSE

Formatted: Font: 12 pt

Gaisler, J., Pavlů, L., Nwaogu, C., Pavlů, K., Hejcman, M., Pavlů, V. V., 2019. Long-term effects of mulching, traditional cutting and no management on plant species composition of improved upland grassland in the Czech Republic. Grass and Forage Science 74, 463–475. https://doi.org/10.1111/gfs.12408

Formatted: Font: 12 pt

Gardiner, T., Hassall, M., 2009. Does microclimate affect grasshopper populations after cutting of hay in improved grassland? J Insect Conserv 13, 97–102. https://doi.org/10.1007/s10841-007-9129-y

Georgi, M. M., Fornoff, F., Gärtner, S. M., Neitzel, S., Geist, A., Klein, A. M., 2022. Timing and mulching frequency affected the number of nests of cavity-nesting wasps that hunt for aphids in forest meadows. J Insect Conserv 26, 973–981. https://doi.org/10.1007/s10841-022-00442-y

Georgi, M. M., Gärtner, S. M., Förschler, M. I., Buse, J., Fornoff, F., Ssymank, A., Oelmann, Y., Klein, A. M., 2023. Mulching time of forest meadows influences insect diversity. Insect Conservation and Diversity 16, 368–378. https://doi.org/10.1111/icad.12629

Glöer, P., Meier-Brook, C., 2003. Süsswassermollusken: Ein Bestimmungsschlüssel für die Bundesrepublik Deutschland. Deutscher Jugendbund für Naturbeobachtung, Göttingen.

Fischer, M., Rudmann-Maurer, K., Weyand, A., Stöcklin, J., 2008. Agricultural Land Use and Biodiversity in the Alps: How Cultural Tradition and Socioeconomically Motivated Changes Are Shaping Grassland Biodiversity in the Swiss Alps. Mountain Research and Development 28, 148–155.

Halada, Ľ., David, S., Hreško, J., Klimantová, A., Bača, A., Rusňák, T., Buraľ, M., Vadel, Ľ., 2017. Changes in grassland management and plant diversity in a marginal region of the Carpathian Mts. in 1999–2015. Science of The Total Environment 609, 896–905. https://doi.org/10.1016/j.scitotenv.2017.07.066

Hartig F., 2022. DHARMa: Residual Diagnostics for Hierarchical (Multi-Level / Mixed) Regression Models_. R package version 0.4.6, <https://CRAN.R-project.org/package=DHARMa>.

Formatted: Font: 12 pt

Formatted: Font: 12 pt

Formatted: Font: 12 pt

Formatted: Font: 12 pt

Formatted: Font: 12 pt

Hejcman, M., Hejcmanová, P., Pavlů, V., Beneš, J., 2013. Origin and history of grasslands in Central Europe – a review. Grass and Forage Science 68, 345–363. https://doi.org/10.1111/gfs.12066

Hesslerová, P., Pokorný, J., Brom, J., 2013. Daily dynamics of radiation surface temperature of different land cover types in a temperate cultural landscape: Consequences for the local climate. Ecological Engineering 54, 145–154. https://doi.org/10.1016/j.ecoleng.2013.01.036

Horan, B., Hennessy, D., O’Donovan, M. (Eds.), 2018. Sustainable meat and milk production from grasslands: proceedings of the 27th General Meeting of the European Grassland Federation, Cork, Ireland, 17 - 21 June 2018, Grassland Science in Europe$Ivolume 23. Presented at the European Grassland Federation, Wageningen Academic Publishers, Wageningen.

Humbert, J.--Y., Ghazoul, J., Sauter, G., Walter, T., 2010. Impact of different meadow mowing techniques on field invertebrates. Journal of Applied Entomology 134, 592–599. https://doi.org/10.1111/j.1439-0418.2009.01503.x

Humbert, J.--Y., Ghazoul, J., Walter, T., 2009. Meadow harvesting techniques and their impacts on field fauna. Agriculture, Ecosystems & Environment 130, 1–8. https://doi.org/10.1016/j.agee.2008.11.014

Iqbran, K., 2019. Role of Mulching in Pest Management and Agricultural Sustainability, SpringerBriefs in Plant Science. Springer International Publishing, Cham. https://doi.org/10.1007/978-3-030-22301-4

Janssen, J., Rodwell, J., García Criado, M., Gubbay, S.,., Haynes, T., Nieto, A., Sanders, N., Landucci, F., Loidi, J., Ssymank, A., Tahvanainen, T., Valderrabano, M., Acosta, A., Aronsson, M., Arts, G., Attorre, F., Bergmeier, E., Bijlsma, R. J., Bioret, F., Gubbay, Susan, J., Bioret, F., Biţă-Nicolae, C., Biurrun, I., Calix, M., Capelo, J., Čarni, A., Chytrý, M., Dengler, J., Dimopoulos, P., Essl, F., Gardfjell, H., Gigante, D., Giusso del Galdo, G., Hájek, M., Jansen, J., Kapfer, J., Mickolajczak, A., Molina, J.A., Molnár, Z., Paternoster, D., Piernik,

Formatted: Font: 12 pt

Formatted: Font: 12 pt

Formatted: Font: 12 pt

Formatted: Font: 12 pt

Formatted: Font: 12 pt

A., Poulin, B., Renaux, B., Schaminée, J. H. J., Šumberová, K., Toivonen, H., Tonteri, T., Tsiripidis, I., Tzonev, R., Valachovič, M., 2016. European Red List of Habitats Part 2. Terrestrial and freshwater habitats. Publications Office of the European Union, Luxembourg. https://doi.org/10.2779/091372

Joyce, C. B., Simpson, M., Casanova, M., 2016. Future wet grasslands: ecological implications of climate change. Ecosystem Health and Sustainability 2, e01240. https://doi.org/10.1002/ehs2.1240

Kaštovská, E., Simek, M., Santruckova, H., 2005. Microbial transformation of organic matter in soils of montane grasslands under different management. Applied Soil Ecology 28, 225–235. https://doi.org/10.1016/j.apsoil.2004.08.002

Kerney, M. P., Cameron, R. A. D., Jungbluth-, J. H., 1983. Die Landschnecken Nord- und Mitteleuropas: ein Bestimmungsbuch für Biologen und Naturfreunde. Paul Parey, Hamburg und Berlin.

Klimeš, L., Hájek, M., Mudrák, O., Dančák, M., Preislerová, Z., Hájková, P., Jongepierová, I., Klimešová, J., 2013. Effects of changes in management on resistance and resilience in three grassland communities. Applied Vegetation Science 16, 640–649. https://doi.org/10.1111/avsc.12032

Kuemmerle, T., Hostert, P., Radeloff, V. C., ~~Van Der~~van der Linden, S., Perzanowski, K., Kruhlov, I., 2008. Cross-border Comparison of Post-socialist Farmland Abandonment in the Carpathians. Ecosystems 11, 614–628. https://doi.org/10.1007/s10021-008-9146-z

Lenth, R. (2023). _emmeans: Estimated Marginal Means, aka Least-Squares Means_. R package version 1.8.4-1, <https://CRAN.R-project.org/package=emmeans>.

Liira, J., Issak, M., Jõgar, Ü., Mändoja, M., Zobel, M., 2009. Restoration Management of a Floodplain Meadow and Its Cost-Effectiveness — the Results of a 6-Year Experiment. Annales Botanici Fennici 46, 397–408. https://doi.org/10.5735/085.046.0504

Formatted: Hyperlink, Font: Calibri, 11 pt, No underline, Font color: Auto

Formatted: Font: 12 pt

Formatted: Font: 12 pt

Formatted: Font: 12 pt

Formatted: Font: 12 pt

Formatted: Font: 12 pt

Formatted: Font: 12 pt

~~Marini, L., Klimek, S., Battisti, A., 2011. Mitigating the impacts of the decline of traditional farming on mountain landscapes and biodiversity: a case study in the European Alps. Environmental Science & Policy 14, 258–267. https://doi.org/10.1016/j.envsci.2010.12.003~~

Mašková, Z., Doležal, J., Květ, J., Zemek, F., 2009. Long-term functioning of a species-rich mountain meadow under different management regimes. Agriculture, Ecosystems & Environment 132, 192–202. https://doi.org/10.1016/j.agee.2009.04.002

McArdle, B. H., Anderson, M. J., 2001. Fitting Multivariate Models to Community Data: A Comment on Distance-Based Redundancy Analysis. Ecology 82, 290–297. https://doi.org/10.2307/2680104

Moog, D., Poschlod, P., Kahmen, S., Schreiber, K.~~-~~ F., 2002. Comparison of species composition between different grassland management treatments after 25 years. Applied Vegetation Science 5, 99–106. https://doi.org/10.1111/j.1654-109X.2002.tb00539.x

Newell, ~~PF~~P. F. 1967. Mollusca. In Burgess A, Raw F. (ed) Soil Biology. Academic Press, London, pp. 413-444.

Oksanen J., Simpson G., Blanchet F., Kindt R., Legendre P., Minchin P., O'Hara R., Solymos P., Stevens M., Szoecs E., Wagner H., Barbour M., Bedward M., Bolker B., Borcard D., Carvalho G., Chirico M., De Caceres M., Durand S., Evangelista H., FitzJohn R., Friendly M., Furneaux B., Hannigan G., Hill M., Lahti L., McGlinn D., Ouellette M., Ribeiro Cunha E., Smith T., Stier A., Ter Braak C., Weedon J. 2024. _vegan: Community Ecology Package_. R package version 2.6-8, <https://CRAN.R-project.org/package=vegan>.

Pavlů, L., Gaisler, J., Hejcman, M., Pavlů, V. V., 2016. What is the effect of long-term mulching and traditional cutting regimes on soil and biomass chemical properties, species richness and herbage production in Dactylis glomerata grassland? Agriculture, Ecosystems & Environment 217, 13–21. https://doi.org/10.1016/j.agee.2015.10.026

Formatted: Font: 12 pt

Formatted: Font: 12 pt

Pech, P., Dolanský, J., Hrdlička, R., Leps, J., 2015. Differential response of communities of plants, snails, ants and spiders to long-term mowing in a small-scale experiment. Community Ecology 16, 115–124. https://doi.org/10.1556/168.2015.16.1.13

Formatted: Font: 12 pt

Petermann, J. S., Buzhdygan, O. Y., 2021. Grassland biodiversity. Current Biology 31, R1195–R1201. https://doi.org/10.1016/j.cub.2021.06.060

Pielou, E. C. 1975. Ecological diversity. John Wiley & Sons, New York.

Pižl, V., Starý, J., 2001. The effects of mountain meadows management on soil fauna communities (on example of earthworms and oribatid mites). Silva Gabreta 7, 87–96.

Formatted: Font: 12 pt

Poschlod, P., Wallisdevries, M., 2002. The historical and socioeconomic perspective of calcareous grasslands - Lessons from the distant and recent past. Biological Conservation 104, 361–376. https://doi.org/10.1016/S0006-3207(01)00201-4

Formatted: Font: 12 pt

R Core Team. 2022. R: A language and environment for statistical computing. R Foundation for Statistical Computing, Vienna, Austria. -URL https://www.R-project.org/.

Ryser, P., Langenauer, R., Gigon, A., 1995. Species Richness and Vegetation Structure in a Limestone Grassland after 15 Years Management with Six Biomass Removal Regimes. Folia Geobotanica 30, 157–167. https://doi.org/10.1007/BF02812095

Formatted: Font: 12 pt

Schmitt, T., 2003. Influence of forest and grassland management on the diversity and conservation of butterflies and burnet moths (Lepidoptera, Papilionoidea, Hesperiidae, Zygaenidae). Animal Biodiversity and Conservation 26, 51–67.

Formatted: Font: 12 pt

Shirish, P., Kelkar, S., Bhalerao, S., 2013. Mulching: A Soil and Water Conservation Practice. Research Journal of Agriculture and Forestry Sciences 1, 26-29.

Squires, V. R., Dengler, J., Hua, L., Feng, H., 2018. Grasslands of the World: Diversity, Management and Conservation. CRC Press.

Steidle, J. L. M., Kimmich, T., Csader, M., Betz, O., 2022. Negative impact of roadside mowing on arthropod fauna and its reduction with ‘arthropod-friendly’ mowing technique. Journal of Applied Entomology 146, 465–472. https://doi.org/10.1111/jen.12976

~~Stoate, C., Báldi, A., Beja, P., Boatman, N., Herzon, I., Doorn, A., Snoo, G.R., Rakosy, L., Ramwell, C., 2009. Ecological Impacts of Early 21st Century Agricultural Change in Europe – A Review. Journal of environmental management 91, 22–46. https://doi.org/10.1016/j.jenvman.2009.07.005~~

Szentes, O., 2023. Szárazság Magyarországon 2022-ben és a múltban. - Drought in Hungary in 2022 and the past. Légkör 68, 9–19.

Theenhaus A, Scheu S ~~(~~., 1996~~)~~. The influence of slug (*Arion rufus*) mucus and cast material addition on microbial biomass, respiration, and nutrient cycling in beech leaf litter. Biol Fertil Soils 23:80–85. https://doi.org/10.1007/BF00335822

Wehner, K., Renker, C., Brückner, A., Simons, N., Weisser, W., Blüthgen, N., 2019. Land-use in Europe affects land snail assemblages directly and indirectly by modulating abiotic and biotic drivers. Ecosphere 10, e02726. https://doi.org/10.1002/ecs2.2726

Welter-Schultes, F. W., 2012. European non-marine molluscs, a guide for species identification. Planet Poster Editions, Göttingen.

~~Zechmeister, H., Schmitzberger, I., Steurer, B., Peterseil, J., Wrbka, T., 2003. The influence of land-use practices and economics on plant species richness in meadows. Biological Conservation 114, 165–177. https://doi.org/10.1016/S0006-3207(03)00020-X~~

~~Zelený, David, Štaritová, Daniela, Mašková, Zuzana, Květ, Jan, 2001. Management effects on a mountain meadow plant community. Silva Gabreta 7., 45–54.~~

Appendix

Formatted: Font: 12 pt

Formatted: Font: 12 pt

Formatted: Font: 12 pt

	Pretreatment			Posttreatment		
	Control	Mowed	Mulched	Control	Mowed	Mulched
Szilvásvárad	260	280	193	239	117	253
Tarnalelesz	559	903	877	447	510	595

Appendix 1. Total number of living individuals in different treatment parcelsplots and periods in the two study area.

	Pretreatment			Posttreatment		
	Control	Mowed	Mulched	Control	Mowed	Mulched
Szilvásvárad	260	280	193	239	117	253
Tarnalelesz	559	903	877	447	510	595

Appendix 2. Pairwise comparison of treatments before the treatments. Posthoc tests from GLMMs separately fitted to each response variable (see the main text for details).

	Pretreatment		
Response variable	Control-Mowed	Control-Mulched	Mowed-Mulched
Number of live specimens	z = -1.39, p = 0.35	z = -0.66, p = 0.79	z = 0.75, p = 0.73
Number of species	t = -2.20, p = 0.07	t = -0.27, p = 0.96	t = 1.92, p = 0.14
Shannon diversityEvenness	t = 0.50, p = 0.87	t = -0.56, p = 0.84	t = -1.09, p = 0.52

Formatted: Centered

Formatted: No widow/orphan control

Formatted Table

Formatted: No widow/orphan control

Formatted: No widow/orphan control

Formatted: No widow/orphan control

Formatted: No widow/orphan control

Appendix 2. Pairwise comparison of treatments before the treatments. Posthoc tests from GLMMs separately fitted to each response variable (see the main text for details).

Response variable	Control	Mowed	Mulched
Number of live specimens (pre-/post-treatment)	z = 1.62, p = 0.10	**z = 4.68, p < 0.0001**	z = 1.43, p = 0.15
Number of species (pre-/post-treatment)	t = 1.53, p = 0.13	**t = 5.86, p < 0.0001**	t = 1.62, p = 0.11
Shannon diversity (pre-/post-treatment)	t = -0.31, p = 0.76	**t = -2.39, p = 0.02**	t = 1.17, p = 0.24

Appendix 3. Changes occurring in different treatment parcelsplots due to treatment. Posthoc tests from GLMMs separately fitted to each response variable (see the main text for details). Legend: Bold font indicates significant differences.

Response variable	Control	Mowed	Mulched
Number of live specimens (pre-/post-treatment)	z = 1.62, p = 0.10	**z = 4.68, p < 0.0001**	z = 1.43, p = 0.15
Number of species (pre-/post-treatment)	t = 1.53, p = 0.13	**t = 5.86, p < 0.0001**	t = 1.62, p = 0.11

Evenness (pre-/post-treatment)	t = -0.31, p = 0.76	**t = -2.39, p = 0.02**	t = 1.17, p = 0.24

Editor's decision

Thank you for submitting your manuscript to PLOS ONE. After careful consideration, we feel that it has merit but does not fully meet PLOS ONE's publication criteria as it currently stands. The study addresses an important and underexplored topic but requires substantial improvements in its methodology, data analysis, and presentation. Therefore, we invite you to submit a revised version of the manuscript that addresses the points raised during the review process.

Please submit your revised manuscript by Jan 20 2025 11:59PM. If you will need more time than this to complete your revisions, please reply to this message or contact the journal office at plosone@plos.org. When you're ready to submit your revision, log on to https://www.editorialmanager.com/pone/ and select the 'Submissions Needing Revision' folder to locate your manuscript file.

Please include the following items when submitting your revised manuscript:

- A rebuttal letter that responds to each point raised by the academic editor and reviewer(s). You should upload this letter as a separate file labeled 'Response to Reviewers'.
- A marked-up copy of your manuscript that highlights changes made to the original version. You should upload this as a separate file labeled 'Revised Manuscript with Track Changes'.
- An unmarked version of your revised paper without tracked changes. You should upload this as a separate file labeled 'Manuscript'.

Additional Editor Comments:

The study's limited scale (two sites, three treatments) necessitates a careful acknowledgement of its constraints.

Thank you for your comment, we have included your suggestion in the Discussion chapter.

Include limitations of the study and suggestions for future research, particularly regarding long-term effects and additional taxa.

We have made the requested additions int he Discussion chapter..

Provide precise details about the mowing and mulching machinery used (e.g., bar mower, disc mower, or flail mulcher).

The parameters were defined in the Method section upon your request.

Address awkward phrasing and inconsistent terminology (e.g., replace "parcel" with "plot," and "vegetable origin" with "organic material").

Based on your and the reviewers' helpful and detailed suggestions, we have made several modifications to the wording of our manuscript. We are confident that the readability of the manuscript has improved.

Differentiate between immediate and longer-term effects of management practices and contextualize results within this framework.

We have increased the elaboration of the question in both the Introduction and the Discussion chapters.

[Note: HTML markup is below. Please do not edit.]

Reviewers' comments:

Reviewer's Responses to Questions

Comments to the Author

1. Is the manuscript technically sound, and do the data support the conclusions?

The manuscript must describe a technically sound piece of scientific research with data that supports the conclusions. Experiments must have been conducted rigorously, with appropriate controls, replication, and sample sizes. The conclusions must be drawn appropriately based on the data presented.

Reviewer #1: Yes

Reviewer #2: Yes

---

2. Has the statistical analysis been performed appropriately and rigorously?

Reviewer #1: Yes

Reviewer #2: Yes

---

3. Have the authors made all data underlying the findings in their manuscript fully available?

The PLOS Data policy requires authors to make all data underlying the findings described in their manuscript fully available without restriction, with rare exception (please refer to the Data Availability Statement in the manuscript PDF file). The data should be provided as part of the manuscript or its supporting information, or deposited to a public repository. For example, in addition to summary statistics, the data points behind means, medians and variance measures should be available. If there are restrictions on publicly sharing data—e.g. participant privacy or use of data from a third party—those must be specified.

Reviewer #1: Yes

Reviewer #2: No

---

4. Is the manuscript presented in an intelligible fashion and written in standard English?

PLOS ONE does not copyedit accepted manuscripts, so the language in submitted articles must be clear, correct, and unambiguous. Any typographical or grammatical errors should be corrected at revision, so please note any specific errors here.

Reviewer #1: No

Reviewer #2: Yes

---

5. Review Comments to the Author

Please use the space provided to explain your answers to the questions above. You may also include additional comments for the author, including concerns about dual publication, research ethics, or publication ethics. (Please upload your review as an attachment if it exceeds 20,000 characters)

Reviewer #1: This study investigates the effects of two different grassland management techniques—mowing and mulching—on mollusk communities in two wet meadow sites. The aim was to determine which management practice would have the least detrimental impact on these communities. While not explicitly stated, the authors anticipated that the management practices would have varying effects on the number of live mollusk specimens, species richness, and Shannon diversity. The authors found that mowing have significant effect on the mollusk communities: mowing reduced the number of living individuals and number of species, but increased Shannon diversity. Mulching had no significant effect. The study concludes that grassland management practices can alter mollusk communities in wet meadows, highlighting the importance of considering these impacts when choosing management strategies.

The authors' experimental design for this field study is commendable. Despite the small scale, the research addresses a relevant question regarding conservation management, particularly given the scarcity of literature on the effects of mulching on grassland fauna. A key weakness of the study design is the inconsistency in sampling times between years. While pre-treatment data were collected in June, post-treatment data were collected in August of the following year.

Thank you for your comment. The pre-management data collection, mechanical management and post-treatment data collection were performed simultaneously in the control, mowed and mulched plots. Consequently, the same amount of time elapsed between each action in all three treatment plots, therefore we believe that the results of our tests (a significant interaction between treatment and sampling period) were not affected by the fact that it covered a period of 14 months rather than a calendar year. We agree that the terms "short term" or "one year"

used for the duration of our work are not accurate, so we have corrected this in the manuscript.

The study's short-term before-after design, without subsequent monitoring in the years following treatment application, restricts its ability to assess long-term community responses and potential delayed effects of mowing and mulching.

Our design was specifically to examine the short-term effects of treatments. We tried to avoid to draw long-term conclusions based on our results. Changes occurring in the short term may help to identify adverse processes early, which allows for the transition to other, more appropriate methods in sensitive areas. We also described this idea in the manuscript.

While the manuscript generally meets the formal requirements of a scientific paper, the language requires substantial improvement. Numerous typos, awkward phrasing, and occasional illogical sentence structures suggest potential translation issues.

We are very grateful that you devoted so much energy to improving the language of our manuscript! Your suggestions meant a lot to us, so we used them in almost every case to improve the manuscript. We also asked a fluent English speaker to improve the language of our MS.

Abstract:
line19: ...maintained through appropriate management method …
line21: shredding, instead of fragmentation
line22: ...is becoming more widely practiced. Or something similar
line22-23: Informal. It could be replaced with more precise academic language.
line30-31: ...while also preserving their biodiversity.

Introduction:
line42: use in-text reference consistently. Suggestion: Squires et al. 2018
line40-41: The phrase "necessary for, first the nomadic lifestyle of livestock grazing" is awkwardly phrased and could be restructured for smoother readability.
line43-44: suggest: since the agricultural revolution, primarily due to human activities
line62: utilizing it, rather than farming
line64-65: Rephrase this sentence, expressions and grammar!
Line73-74: Consider breaking it into two sentences for clarity.
Line76-78: I do not think such explanation is needed for the practices.
Line79-81: Suggestion: The encroachment of shrubs and woody plants threatens the persistence of grasslands and can also negatively impact farming activities.
Line81: A striking example of these changes can be seen in Central and Eastern Europe…
Line85: use ; in the parentheses between 2 references
Line86: consistency in in-text references still needed
Line88: definitions, rather than meanings
Line96: lawn is a wrong translation of grassland/meadow
Line100: rather an area within a few years
Line109: „Less favourable temperatures" could be more specific, mentioning higher

temperatures
Line114: Organic material/Biomass
Line132: ...has been less extensively studied.
Line124: do not use the word „farmed” in this context
Line151-152: The phrase "At most, conclusions can be drawn from experience with one kind of mulching" sounds awkward and informal.
Line155: populations can’t be indicator species
Line156: "to say after the first year" could be more formal
Line158-159: Suggest: We selected undisturbed wet meadows that had remained untouched for decades and characterized their snail communities. We then mowed and mulched portions of these areas using standard agricultural machinery, following a stratified experimental design.

In the aims section, you could be more precise, mention at least hte factors, which you use to assess how „harmful” these managements are. Hypotheses could be clarified as well.

Thank you for your comment, we have improved the sections indicated above according to your request.

Materials and methods:
Line175: I would avoid the phrasing „without any obvious microhabitat structure”, as microhabitat structure depends of the definition. Be precise.
Line177: „for the last 20-30 years” 10 years matters a lot!
Line180: Figure 1.: In my opinion, the two smaller maps below are not very informative. Consider replacing them with a different figure. It's important to show the location within Hungary, but additionally, it might be useful to show Hungary's location within Europe, rather than using two other small-scale maps which shows nothing. Including satellite images to illustrate vegetation coverage would be helpful, and adding some toponyms could assist those who want to identify the study sites. Alternatively, you could completely reorganize the figure. Besides the map of Hungary, photographs of the meadows might be more useful for better understanding.

We have transformed the figure. We considered the photographic solution to be appropriate, which was indeed more informative than the original. We solved the identification of the locations using coordinates given in the Method section.

Line196: It would be more obvious to show 45 sampling points on Figure 2, rather than a grid.

The figure has been modified and incorporated into Figure 1.

Line204: don’t need „once”
Line207-208: I think the aouthors did not mimic the traditional mowing of hay this way. The cut plant material remaines on the are for some days for drying. This is a totally different effect on snails, then removing the plant material immediately.

Thank you for your comment. To clarify the issue, we have explained the difference between the treatment methods we use and the traditional ones in the Discussion section. It is interesting that the articles based on mowing experiments only mention the fact of "mowing". After cutting the vegetation, they do not detail how many sub-steps were performed (drying, turning, windrowing, baling, etc.) until the cut biomass was removed. Perhaps more emphasis should be placed on detailing these in future studies. In our modification we also highlighted that management practice of nature conservation areas by mowing resembles more to our treatment than traditional hay producing mowing.

Line222: „vegetable origin":)
Line241: don't need „interactive"
Line253: don't need „however"

Results:
Line276: scientific names should be italic in Table 1
Line292: authors may use Shannon diversity consitently
Line305-306: indexing $\chi 21$ $\chi 22$ $\chi 22$
On Figure 3c, relocate y axis title

Discussion:
Line328: check this sentence
Line 389: there is in additional comma in the reference parentheses

I would like to read some limitations, possible future prospects in the discussion. This part was much better formulated than the other parts of the article.

The indicated parts were corrected in the manuscript at your request.

Conclusion:
Line403: avoid such strong emotional phrases like „clearly evidendt"
Line405-406: Be cautious with the word „ptactical". Highlight the exact result like:...without the negative effects of mowing on snail communities
Line407: „to maintain" and „to preserve" would fit better
Line407-408: Link it more directly to your findings. Like: „Given the observed negative effects of mowing…"
Line413-415: This is too general. Connect it to the specific ecological aspects revealed by the study.

We modified this part at your request.

Literature:
Again, be consistent. Corrected author initials spacing (e.g., "KJ." → "K. J."), it is used multiple ways.

Thank you for your comment. We checked and corrected spacings throught the whole manuscript.

Appendix 2: Format the first column, odd spacing.

We modified this part at your request.

Reviewer #2: The manuscript „ Experimental comparison of mowing and mulching on snail communities in wet meadows“ by Farkas et al. studied the effect of two common grassland management methods on the number of individuals, the species numbers and the diversity (Shannon-Index) of snails in wet meadows in Hungary one year after the treatment.
While mulching, the shredding of vegetation and leaving it on the ground, had no effect, mowing reduced the number of individuals and species, but increased the Shannon-diversity. From the most abundant species, only one was significantly affected by the treatment, with an increase in the number of individuals after mulching.
Technically, the study seems to be sound and the results seem to be well supported by the data. Samples were collected with appropriate methods and the data were analysed using up-to-date statistical methods. Therefore, considering the lack of data on the effect of intensive agriculture on snails in the face of the current biodiversity crisis the data certainly deserve publication.
My main concern is that the analysis of data is a bit meager and that the study is relatively small and consisted of one experiment only with three treatments (control, mowing, mulching) on two sites. While the first aspect can be addressed by some more statistical analyses (see remarks below), the second cannot be changed. Therefore, I would leave it to the editor to decide if this is sufficient to accept the manuscript for publication. Apart from this, I have several aspects that should be addressed to make the manuscript suitable for publication.

We thanks the reviewer effort on our MS and reply her/his detailed comments below.

Detailed comments
1. While it is interesting that only mowing affected individual numbers, species numbers and Shannon-diversity, it would be interesting to know the underlying reasons. This is the case for the demonstrated effects of mowing, but also the absence of any effect of mulching.

We now highlight some reasons why mowing had and mulching had not have an effect on snail communities. These are basically connected to the fact that after mowing all cut material was removed from the area, while after mulching the material remained which prevented the immediate desiccation of the area.

For instance, the unexpected rise in the Shannon-diversity by mowing is explained by the authors by a potential decline of individual numbers of abundant species and the associated increase of evenness.

Here we have to point out a mistake we made in the previous version of the MS: We actually calculated Pileou’s evenness index for our samples which we incorrectly called the Shannon diversity. We now corrected this naming error throughout the MS.

This is an interesting hypothesis which could (and should) be tested by the authors with their data. First, authors should calculate the evenness for their samples. In addition, a closer look at the data should reveal whether there are in fact abundant species which have decreased in individual numbers.

We calculated the decrease in abundance as a function of original (pretreatment) abundance and got a negative relationship between the two in each treatment. Unfortunately, the distributional properties of our data prevented us to statistically test if this negative slope was steeper for the mowing than for the other two treatments. Therefore, we just present our idea as a hypothesis in the Discussion.

Likewise, it would be interesting to know, whether mulching really had no impact on the snail community (as suggested by the numbers of individuals and species), or whether there might have been a species turn-over between the plots before and after the treatment. This could be analysed using beta-diversity and a similarity analysis. The same analysis should be done with the data from the mown plots. In addition, authors should also look at the species lists from the different treatments and the ecology of species that have changed in abundance. In conclusion, a more detailed analysis of the data could over insights into the mechanisms which are responsible for the observed effects.

Thank you for this suggestion. We took a broader approach in the current version of the MS and use PERMANOVA (adonis2 function of the vegan R package) to test if our terms (treatment, sampling and their interaction) have any effect on the overal compositions of the differently treated plots. We got the same result as previously, namely we have a significant interaction between treatment and sampling which indicates that the communities reacted differently to the different treatments over the sampling period. Examing the estimated parameters of the PERMANOVA indicates that this differences only significant in the case of mowing in accordance with our previous results.

2. While it is correct that the authors have included “site” as random variable in their model, it would be also interesting to learn whether the observed effects occur equally on both sites. Therefore, I suggest an analysis in which site is used as fixed factor.

We repeated all of our analyses with including site as a fixed factor and none of our conclusion changed so we retained the original analyses.

3. Generally, the manuscript is well readable but not very well written and, in some parts, not very stringent and wordy. This is especially true for the introduction and the discussion. Also, the English sometimes seems wrong to me. For instance, the term “parcel” is used for the German term “Parzelle”. I think the correct translation should be “plots”. I have made a number of suggestions in the attached pdf-File, but I am not a native speaker. Therefore, I strongly recommend proof reading of the manuscript by a native speaker experienced in scientific writing before submission of the revised version.

At your request, we had the manuscript re-checked for grammatical accuracy by a professional English-speaking researcher.

4. Authors should not refer to snails as indicator species in the context of the study. As mentioned by the authors themselves, different taxa react very differently to agricultural management measures. Snaily might be indicators for themselves, but not for other groups!

Thank you for your comment, we have made the necessary changes in the Intoduction chapter.

5. Authors should be more specific with respect to the effect of mowing or mulching on the target organisms. This refers to line 133 of the introduction, but also to the rest of the manuscript. In the available literature, the effect of mowings has been studied immediately after the treatment, sometime after the treatment (weeks or years), or during longer periods of time. While effects found immediately after the treatment have to (and can) be attributed directly to the mowing event itself, i.e. the mortality caused by the mowing machine, effects after weeks or years can be increasingly indirect, e.g. increased mortality due to unfavourable environmental conditions or increased predation of exposed organisms. Authors should explicitly point out that their study refers to the second effects

We modified this part in the Introduction and Discussion chapters.

6. On how many replicates per treatement are the data based. Is it n=15 per site, i.e. n=30 per treatment for the whole study?

We have now clarified it in the Methods section, we have 30 replicate per treatment.

7. Throughout the ms: Tables description should be on top of the table.

We have modified it.

Line 93ff: What is the difference between these two variants of mulching. To me, it sounds identical. Grass is mown and left on the spot. Or is the fact that grass is shredded in variant 3, but not in variant 2?

Thank you for your comment, we made it clear in the Introduction chapter.

Line 207: Please include the type of mowing machine used? Bar mower, Disc mover
Flail mulcher? Conditioner?

We have clarified it in the Method chapter.

Table 2: Use italics for species and genus names

We have modified it in all cases

6. PLOS authors have the option to publish the peer review history of their article (what does this mean?). If published, this will include your full peer review and any attached files.

If you choose "no", your identity will remain anonymous but your review may still be made public.

Do you want your identity to be public for this peer review? For information about this choice, including consent withdrawal, please see our Privacy Policy.

Reviewer #1: No

Reviewer #2: **Yes:** Johannes Steidle

[NOTE: If reviewer comments were submitted as an attachment file, they will be attached to this email and accessible via the submission site. Please log into your account, locate the manuscript record, and check for the action link "View Attachments". If this link does not appear, there are no attachment files.]

While revising your submission, please upload your figure files to the Preflight Analysis and Conversion Engine (PACE) digital diagnostic tool, https://pacev2.apexcovantage.com/. PACE helps ensure that figures meet PLOS requirements. To use PACE, you must first register as a user. Registration is free. Then, login and navigate to the UPLOAD tab, where you will find detailed instructions on how to use the tool. If you encounter any issues or have any questions when using PACE, please email PLOS at figures@plos.org. Please note that Supporting Information files do not need this step.

In compliance with data protection regulations, you may request that we remove your personal registration details at any time. (Remove my information/details). Please contact the publication office if you have any questions.
