# Supplementary material for: The effect of mowing and mulching on snail communities: an experiment in wet meadows
Source: PLoS One. 2025 Jul 10;20(7):e0314670. doi: 10.1371/journal.pone.0314670 (PMC12244540; doi:10.1371/journal.pone.0314670)
Supplement: Attachment — Submitted filename: Response_to Reviewers.docx [file pone.0314670.s008.docx]

Dear Editors,

You will find our responses to your requests and suggestions in the appropriate places, in blue font.

Dear Dr. Farkas,

Thank you for submitting your manuscript to PLOS ONE. After careful consideration, we feel that it has merit but does not fully meet PLOS ONE’s publication criteria as it currently stands. Therefore, we invite you to submit a revised version of the manuscript that addresses the points raised during the review process.

Although you thoroughly revised your manuscript, both reviewers found further room for improvement.

Please submit your revised manuscript by Jul 27 2025 11:59PM. If you will need more time than this to complete your revisions, please reply to this message or contact the journal office at [plosone@plos.org](mailto:plosone@plos.org). When you're ready to submit your revision, log on to https://www.editorialmanager.com/pone/ and select the 'Submissions Needing Revision' folder to locate your manuscript file.

Please include the following items when submitting your revised manuscript:

- A rebuttal letter that responds to each point raised by the academic editor and reviewer(s). You should upload this letter as a separate file labeled 'Response to Reviewers'.
- A marked-up copy of your manuscript that highlights changes made to the original version. You should upload this as a separate file labeled 'Revised Manuscript with Track Changes'.
- An unmarked version of your revised paper without tracked changes. You should upload this as a separate file labeled 'Manuscript'.

If you would like to make changes to your financial disclosure, please include your updated statement in your cover letter. Guidelines for resubmitting your figure files are available below the reviewer comments at the end of this letter.

If applicable, we recommend that you deposit your laboratory protocols in protocols.io to enhance the reproducibility of your results. Protocols.io assigns your protocol its own identifier (DOI) so that it can be cited independently in the future. For instructions see: <https://journals.plos.org/plosone/s/submission-guidelines#loc-laboratory-protocols>. Additionally, PLOS ONE offers an option for publishing peer-reviewed Lab Protocol articles, which describe protocols hosted on protocols.io. Read more information on sharing protocols at <https://plos.org/protocols?utm_medium=editorial-email&utm_source=authorletters&utm_campaign=protocols>.

We look forward to receiving your revised manuscript.

Kind regards,

Edvard Mizsei
Academic Editor
PLOS ONE

Journal Requirements:

Please review your reference list to ensure that it is complete and correct. If you have cited papers that have been retracted, please include the rationale for doing so in the manuscript text, or remove these references and replace them with relevant current references. Any changes to the reference list should be mentioned in the rebuttal letter that accompanies your revised manuscript. If you need to cite a retracted article, indicate the article’s retracted status in the References list and also include a citation and full reference for the retraction notice.

Dear Journal Editors,

As requested, we checked the manuscript reference list. We found two references that were in the text but missing from the reference list, which we have now replaced. The two references were: Cameron and Pokryszko, 2005 and Zedler and Kercher, 2005. Thank you for bringing this to our attention.

[Note: HTML markup is below. Please do not edit.]

Reviewers' comments:

Reviewer's Responses to Questions

**Comments to the Author**

1. If the authors have adequately addressed your comments raised in a previous round of review and you feel that this manuscript is now acceptable for publication, you may indicate that here to bypass the “Comments to the Author” section, enter your conflict of interest statement in the “Confidential to Editor” section, and submit your "Accept" recommendation.

Reviewer #1: All comments have been addressed

Reviewer #2: All comments have been addressed

2. Is the manuscript technically sound, and do the data support the conclusions?

The manuscript must describe a technically sound piece of scientific research with data that supports the conclusions. Experiments must have been conducted rigorously, with appropriate controls, replication, and sample sizes. The conclusions must be drawn appropriately based on the data presented.

Reviewer #1: Yes

Reviewer #2: Yes

3. Has the statistical analysis been performed appropriately and rigorously?

Reviewer #1: Yes

Reviewer #2: Yes

4. Have the authors made all data underlying the findings in their manuscript fully available?

The [PLOS Data policy](http://www.plosone.org/static/policies.action#sharing) requires authors to make all data underlying the findings described in their manuscript fully available without restriction, with rare exception (please refer to the Data Availability Statement in the manuscript PDF file). The data should be provided as part of the manuscript or its supporting information, or deposited to a public repository. For example, in addition to summary statistics, the data points behind means, medians and variance measures should be available. If there are restrictions on publicly sharing data—e.g. participant privacy or use of data from a third party—those must be specified.

Reviewer #1: Yes

Reviewer #2: No

5. Is the manuscript presented in an intelligible fashion and written in standard English?

PLOS ONE does not copyedit accepted manuscripts, so the language in submitted articles must be clear, correct, and unambiguous. Any typographical or grammatical errors should be corrected at revision, so please note any specific errors here.

Reviewer #1: Yes

Reviewer #2: Yes

6. Review Comments to the Author

Please use the space provided to explain your answers to the questions above. You may also include additional comments for the author, including concerns about dual publication, research ethics, or publication ethics. (Please upload your review as an attachment if it exceeds 20,000 characters)

Reviewer #1: The revised manuscript shows clear improvement, and it is evident that the authors have carefully addressed the reviewers’ earlier comments. The text reads more smoothly, the structure and clarity have been improved, and the scientific content is now better communicated. I appreciate the thoughtful revisions and the effort made to respond comprehensively to the feedback. Below are some further suggestions for fine-tuning the manuscript.

27-28: I would highlight the relevance in conservation, e.g.: Therefore, mulching may be a promising candidate for conservation management in wet meadow habitats.

51-52: The end of this sentence is not clear. Should use something to clarify, like ....making this a critical conservation concern.

65: modern?

73: I would insert "management" before strategy.

170: In this case, you should write out the English name of the species in my opinion, or just the scientific name, without parentheses.

172: I understand, what would you like to say with this sentence, but it is formulation isn't clear. Something like "Both sites were flat and appeared to have a homogeneous vegetation structure." would be clear.

194: You don't need the "." after years.

209: The "X" is capitalised in 25x25, change it please.

314-315: Clunky sentence sructure. I would rather choose this: Only for Vertigo angustior did the treatments have significantly different effects on abundance (interaction: ?22 = 7.90, p = 0.019);

326: ...but not in mulched ones,

400: no need for ","

On Figure 2, the species names should be written with italic in my opinion.

Reviewer #2: Generally, the manuscript was improved a lot although it is still very wordy and repetitious. I have made some suggestions in the attached pdf and I think that including these corrections, it is suitable for publication.

Dear Reviewers,

Thank you for your valuable comments that contributed to the development of our manuscript. In the second round of review, both of you made further suggestions for improving the language and style of the manuscript. In each case, your suggestions were justified and important. We have accepted all of your comments and modified the manuscript. Thank you again for your contribution. The work of both of you has greatly helped us to formulate our results in the appropriate scientific form and style.

7. PLOS authors have the option to publish the peer review history of their article ([what does this mean?](https://journals.plos.org/plosone/s/editorial-and-peer-review-process#loc-peer-review-history)). If published, this will include your full peer review and any attached files.


If you choose “no”, your identity will remain anonymous but your review may still be made public.


**Do you want your identity to be public for this peer review?** For information about this choice, including consent withdrawal, please see our [Privacy Policy](https://www.plos.org/privacy-policy).

Reviewer #1: No

Reviewer #2: No

[NOTE: If reviewer comments were submitted as an attachment file, they will be attached to this email and accessible via the submission site. Please log into your account, locate the manuscript record, and check for the action link "View Attachments". If this link does not appear, there are no attachment files.]

While revising your submission, please upload your figure files to the Preflight Analysis and Conversion Engine (PACE) digital diagnostic tool, <https://pacev2.apexcovantage.com/>. PACE helps ensure that figures meet PLOS requirements. To use PACE, you must first register as a user. Registration is free. Then, login and navigate to the UPLOAD tab, where you will find detailed instructions on how to use the tool. If you encounter any issues or have any questions when using PACE, please email PLOS at [figures@plos.org](mailto:figures@plos.org). Please note that Supporting Information files do not need this step.

*In compliance with data protection regulations, you may request that we remove your personal registration details at any time.* [*(Remove my information/details)*](https://www.editorialmanager.com/pone/login.asp?a=r)*. Please contact the publication office if you have any questions.*
